# Supplementary material for: Barley sodium content is regulated by natural variants of the Na+ transporter HvHKT1;5
Source: Commun Biol. 2020 May 22;3:258. doi: 10.1038/s42003-020-0990-5 (PMC7244711; doi:10.1038/s42003-020-0990-5)
Supplement: Supplementary file 1 — Supplemental Information [file 42003_2020_990_MOESM1_ESM.pdf]

Supplementary Information: Natural variants of *HvHKT1;5* regulate sodium content in barley.

Supplementary Figures.

Supplementary Figure 1. Current-voltage (I/V) curve of *X. laevis* oocytes expressing allelic variants of HvHKT1;5. **A**, High Na^+ allele HvHKT1;5N57S. **B**, High Na^+ allele HvHKT1;5V416I. **C,** High Na^+ allele HvHKT1;5S438N. **D,** Low Na^+ allele HvHKT1;5I416V. **E**, Low Na^+ allele Viivi (Na^+_{HAP2}). Currents were recorded in 1 mM, 30 mM of Na^+ or 30 mM K^+ glutamate; data represented in mean ± SEM. n= 3-6.

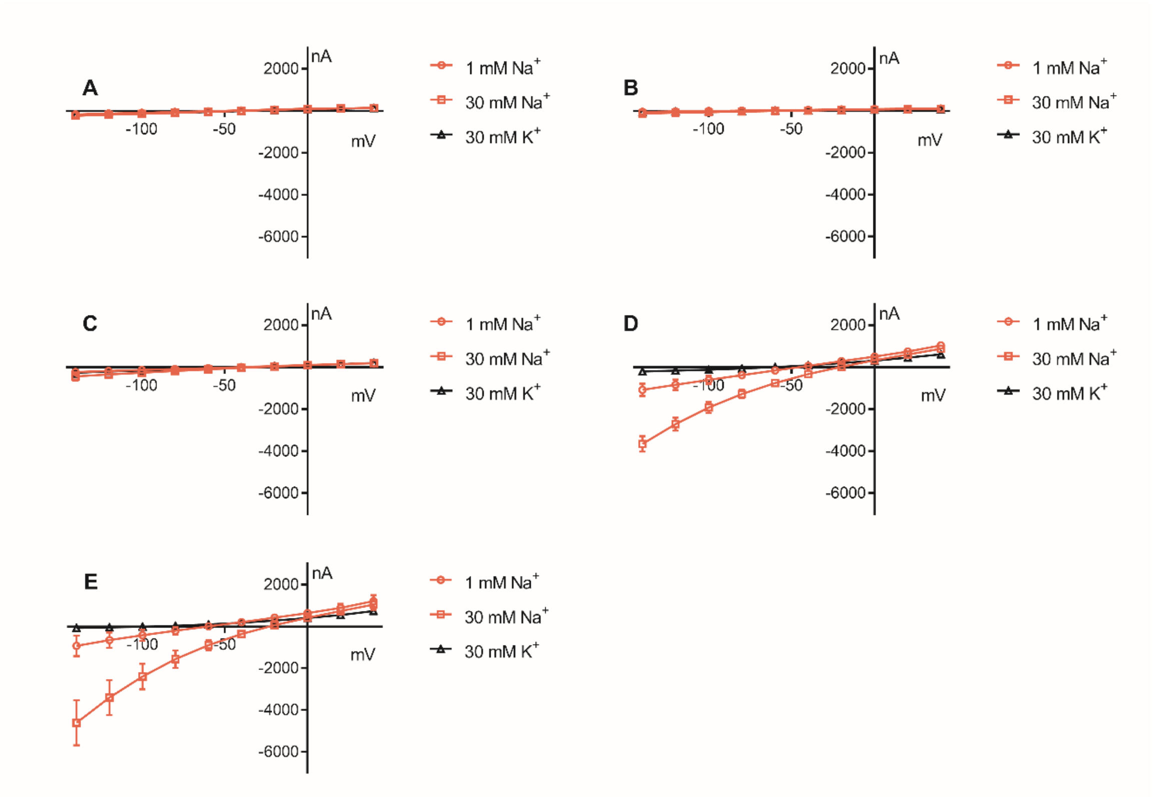

Supplementary Figure 2: Molecular models of HvHKT1;5$_{HAP3_L189}$ and HvHKT1;5$_{HAP3_P189}$ transporters in complex with Na^{+}. a., b. Cartoon representations of HvHKT1;5$_{HAP3_L189}$ (left, grey) and HvHKT1;5$_{HAP3_P189}$ (right, deep teal) with cylindrical α-helices illustrate 3D folds. Constrictions in selectivity filters are bound by four residues (cpk magenta sticks, regular types) that contain Na^{+} (violet spheres). Black arrows illustrate directional flows of Na^{+} that are likely to enter the permeation trajectory by-passing selectivity filter constrictions. Variant residues N57, V416, S438 and L189 (cpk sticks and dots) in HvHKT1;5$_{HAP3_I189}$, and N57, V416 and S438 and P189 (cpk sticks and dots) in HvHKT1;5$_{HAP3_P189}$ are indicated; the dots illustrate volumes of van der Waals radii. Four variations N57, V416 and S438 (regular types), and L189 or P189 (bold types) are shown in HvHKT1;5$_{HAP3}$ proteins, from which P189 is deemed to be critical for protein structure that underlies function. **c., d.** Detailed views of α-helices, which neighbour constrictions of selectivity filters containing Na^{+} that are crucial for permeation function. Na^{+} (violet spheres) are located near the selectivity filter residues S76, G232, G351, G451 (cpk magenta sticks) in HvHKT1;5$_{HAP3}$ structures. In each protein, polar contacts of L189 and P189 (shown in cpk sticks and dots), that are positioned on α-helix 4, are indicated by dashed lines at separations between 2.5 Å and 3.1 Å. Notably, leucine or proline residues in HvHKT1;5$_{HAP3}$ structures effect packing angles of bordering α-helices 4 and 5. This packing angle between α-helices 4 and 5 in HvHKT1;5$_{HAP3_P189}$ is more obtuse (two black arrows pointing to each other).

Supplementary Figure 2

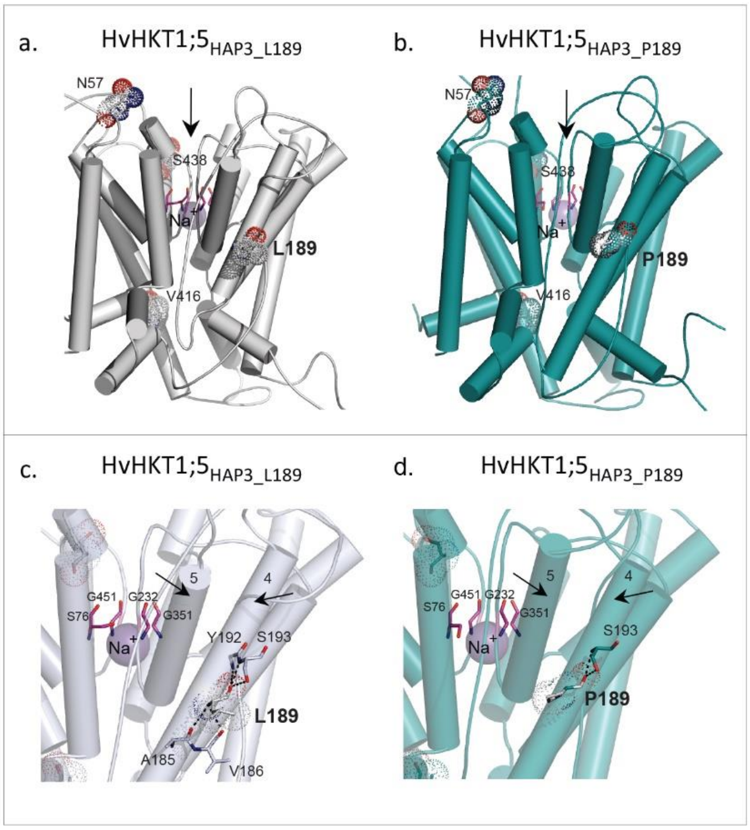

Supplementary Figure 2 Methods.

The most suitable template for HvHKT1;5 transporter proteins was the *B. subtilis* KtrB K+ transporter (Protein Data Bank accession 4J7C, chain I) (Vieira-Pires *et al.*, 2013) as previously identified (Xu *et al.*, 2018). In KtrB, K^+ was substituted by Na^+ during modelling of all HKT1;5 proteins. 3D models of HvHKT1;5$_{HAP3_L189}$ and HvHKT1;5$_{HAP3_P189}$ in complex with Na+ were generated in Modeller 9v19 (Sali and Blundell, 1993) as described previously (Cotsaftis *et al.*, 2012, Waters *et al.*, 2013) incorporating Na^+ ionic radii (Xu *et al.*, 2018) taken from the CHARMM force field (Brooks *et al.*, 2009), on the Linux station running the Ubuntu 12.04 operating system. Best scoring models (from an ensemble of 50) were selected based on the combination of Modeller Objective Function (Shen and Sali, 2006), Discrete Optimised Protein Energy term (Eswar *et al.*, 2008), PROCHECK (Laskowski *et al.*, 1993), ProSa 2003 (Sippl, 1993) and FoldX (Schymkowitz *et al.*, 2005). Structural images were generated in the PyMOL Molecular Graphics System V1.8.2.0 (Schrődinger LLC, Portland, OR, USA). Calculations of angles between selected α-helices in HvHKT1;5 models were executed in Chimera (Pettersen *et al.*, 2004) and evaluations of differences (ΔΔG = ΔGmut-ΔGwt) in Gibbs free energies was performed with FoldX (Schymkowitz *et al.*, 2005). Sequence conservation patterns were analysed with ConSurf (Landau *et al.*, 2005; Celniker *et al.*, 2013) based on 3D models of HvHKT1;5 transporters.

Evaluations of stereo-chemical parameters indicated that the template and HvHKT1;5 models had satisfactory parameters as indicated by Ramachandran plots with two residues positioned in disallowed regions, corresponding to 0.5% of all residues, except of G and P. Average G-factors (measures of correctness of dihedral angles and main-chain covalent bonds) of the template, and the HvHKT1;5$_{HAP3_L189}$ and HvHKT1;5$_{HAP3_P189}$ models, calculated by PROCHECK (0.06, -0.07 and -0.21, respectively), and ProSa 2003 z-scores (measures of Cβ-Cβ pair interactions of -9.0, -5.6 and -6.5, respectively), indicated that template and modelled structures had favourable conformational energies.

Results and Discussion

1. Positional sequence identities between template and target sequences are in the twilight zone (20.6% and 20.4% between the template and HvHKT1;5$_{HAP3_L189}$ and HvHKT1;5$_{HAP3_P189}$ sequences, respectively), emphasising the difficulty of comparative modelling. This indicated that the attention must be paid to sequence alignments to be able to compare 3D models at the structural levels. Three types of alignments were generated, using Muscle (Edgar *et al.*, 2004), MUSTER (Wu and Zhang, 2007) and LOMETS (Wu and Zhang, 2008) algorithms. Input alignments for 3D modelling were generated by the combination of all alignments and secondary structure elements analyses using PsiPred (Buchan *et al.*, 2013), followed by manual adjustments to optimise positions of gaps in alignments.

2. 3D modelling revealed that overall 3D folds were similar, where selectivity filter constrictions carry one serine and three glycine residues, in accordance with their Na^+ ion conductivity (Supplementary Figure 2).

3. Detailed analysed of environments around α-helix 4 and α-helix 5 (two black arrows pointing to each other in Supplementary Figure 2) revealed that L189 in α-helix 4 of

HvHKT1;5_{HAP3_L189} established four polar contacts at separations between 2.7 Å to 3.1 Å with A185, V186, Y192 and S193 neighbouring residues.

4. These extensive polar contacts were not formed in the HvHKT1;5_{HAP3_P189} variant which only established two polar contacts at separations between 2.5 Å to 2.7 Å with S193. The lack of these cooperative binding networks in α-helix 4 around P189, and tight separations may impose severe structural rigidity on 3D folds HvHKT1;5_{HAP3_P189}. These α-helices might no longer properly function in the structural and functional context to ensure Na^+ ion conductance.

5. In HvHKT1;5_{HAP3_L189}, the packing angle between α-helix 4 and α-helix 5 (two black arrows pointing to each other in Supplementary Figure 2) is 9° sharper compared to that in HvHKT1;5_{HAP3_P189} indicating that proline positions affect packing of α-helices in the specific 3D environments of HvHKT1;5 transporters. These changes in structural packing most likely contribute significantly to the structural rigidity and the lack of dynamics in 3D folds during transport.

6. Evaluations of differences of Gibbs free energies of HvHKT1;5_{HAP3} transporters revealed that the L189P mutation was energetically highly unfavourable (highly destabilising), and that the reverse mutation (P189 into L189) restored 100% of this energy loss, as expected.

7. In HvHKT1;5_{HAP3} transporters the differences in Gibbs free energies (ΔΔG) between the P189L variant and the reverse mutation (L189P) were mildly destabilising and similar in both directions, suggesting that the environment of P189 has somewhat adapted to its 3D fold, thus a low level of conductance of Na^+ could be observed in HvHKT1;$5_{HAP3\_P189\_}$.

8. In HvHKT1;5_{HAP3} we identified a positive correlation between structural characteristics of α-helix 4/α-helix 5 (trends in angles based on α-helical planes), differences in Gibbs free energies of forward (P189L) and reverse (L189P) mutations, and the ability to conduct Na^+. This correlation shows that in barley HvHKT1;5 transporters, conservation and variability of specific residues reflect profoundly on the transport function.

9. Sequence conservation patterns, based on 3D models of HvHKT1;5_{HAP3} using 368-370 sequences at sequence identities of 30% and higher (specifications: HMMMER homolog search algorithm, UNIREF-90 Protein database with the E-value cutoff of $1 \cdot 10^{-4}$, Bayesian Model of substitution for proteins), revealed that the P189 variation occurred only in barley.

10. There are four variations (N57, P189, V416 and S438) in HvHKT1;5_{HAP3} compared to HvHKT1;5_{HAP1} that represent 15 testable combinations. Not all of them could be tested for transport function. We suggest that three variations (N57, V416, S438) between HvHKT1;5_{HAP3} and HvHKT1;5_{HAP1} would have a lesser impact on Na^+ conductivity. This is supported by conservation patterns analyses showing that these residues could be substituted by a variety of (mostly hydrophilic) residues, namely to R, S, A, P, G, L, H, D, Y, V, N, T, E, I (for N57), F, L, I, A, T, V (for V416), and N, T, Y, K, Q, S, R, H, A, P (for S438).

References

Brooks BR, Brooks CL 3rd, Mackerell AD Jr, Nilsson L, Petrella RJ, Roux B, Won Y, Archontis G, Bartels C, Boresch S, Caflisch A, Caves L, Cui Q, Dinner AR, Feig M, Fischer S, Gao J, Hodoscek M, Im W, Kuczera K, Lazaridis T, Ma J, Ovchinnikov V, Paci E, Pastor RW, Post CB, Pu JZ, Schaefer M,

Tidor B, Venable RM, Woodcock HL, Wu X, Yang W, York DM, Karplus M (2009) CHARMM: The Biomolecular Simulation Program. J Comp Chem 30, 1545-1615.

Buchan DWA, Minneci F, Nugent TCO, Bryson K, Jones DT (2013) Scalable web services for the PSIPRED protein analysis workbench. Nucleic Acids Res 41, W340-W348.

Celniker G, Nimrod G, Ashkenazy H, Glaser F, Martz E, Mayrose I, Pupko T, Ben-Tal N (2013) ConSurf: using evolutionary data to raise testable hypotheses about protein function. Isr J Chem 53, 199-206

Cotsaftis O, Plett D, Shirley N, Tester M, Hrmova M (2012) A two-staged model of Na+ exclusion in rice explained by 3D modeling of HKT transporters and alternative splicing. PLoS ONE 7, e39865.

Edgar RC (2004) MUSCLE: multiple sequence alignment with high accuracy and high throughput. Nucleic Acids Res 32,1792–1797.

Eswar N, Eramian D, Webb B, Shen MY, Sali A (2008) Protein structure modeling with Modeller, In: Kobe B, Guss M, Huber T (eds) Structural Proteomics. Meth Mol Biol, vol 426. Humana Press.

Landau M, Mayrose I, Rosenberg Y, Glaser F, Martz E, Pupko T, Ben-Tal N (2005) ConSurf 2005: the projection of evolutionary conservation scores of residues on protein structures. Nucleic Acids Res 33, W299-W302.

Laskowski, RA, MW MacArthur, DS Moss, JM Thornton (1993) PROCHECK: a program to check the stereochemical quality of protein structures. J App Crystall 26, 283-291.

Pettersen, EF, Goddard TD, Huang CC, Couch GS, Greenblatt DM, Meng EC, Ferrin TE (2004) UCSF Chimera - A Visualization System for Exploratory Research and Analysis. J Comput Chem 25, 1605-1612.

Sali A, Blundell T (1993) Comparative protein modeling by satisfaction of spatial restraints. J Mol Biol 234, 779-815.

Schymkowitz JWH, F Rousseau, IC Martins, J Ferkinghoff-Borg, F Stricher, Serrano L (2005) Prediction of water and metal binding sites and their affinities by using the Fold-X force field. Proceed Natl Acad Sci USA 102, 10147-10152.

Shen, MY, Sali A (2006) Statistical potential for assessment and prediction of protein structures. Protein Sci 15, 2507-2524.

Sippl MJ (1993) Recognition of errors in three-dimensional structures of proteins. Proteins 17, 355-362.

Vieira-Pires RS, Szollosi A, Morais-Cabral JH (2013) The structure of the KtrAB potassium transporter. Nature 496, 323-328.

Waters S, Gilliham M, Hrmova M (2013) Plant high affinity potassium (HKT) transporters involved in salinity tolerance: structural insights to probe differences in ion selectivity. Int J Mol Sci 14, 7660-7680.

Xu B, Waters S, Byrt CS, Plett D, Tyerman SD, Tester M, Munns R, Hrmova M, Gilliham M (2018) Structural variations in wheat HKT1;5 underpin differences in Na+ transport capacity. Cell Mol Life Sci 75, 1133-1144.

Wu S, Zhang Y (2007) LOMETS: A local meta-threading-server for protein structure prediction. Nucleic Acids Res 35, 3375-3382

Wu S, Zhang Y (2008) MUSTER: Improving protein sequence profile-profile alignments by using multiple sources of structure information. Proteins 72, 547-556.

Supplementary Figure 3: Influence of L189P polymorphism in HvHKT1;5 on grain K^+ accumulation. Mature grain K^+ content after barley accessions were exposed to different levels of NaCl at the fourth leaf stage of development. For boxplots the horizontal bar of the boxplot shows the median, the box delineates the first and third quartile, and the whiskers show + /−1.5 × IQR. White bars indicate 0mM of NaCl, light grey bars indicate 150mM NaCl, and dark grey indicates 250Mm NaCl added to plants.

Supplementary Figure 3

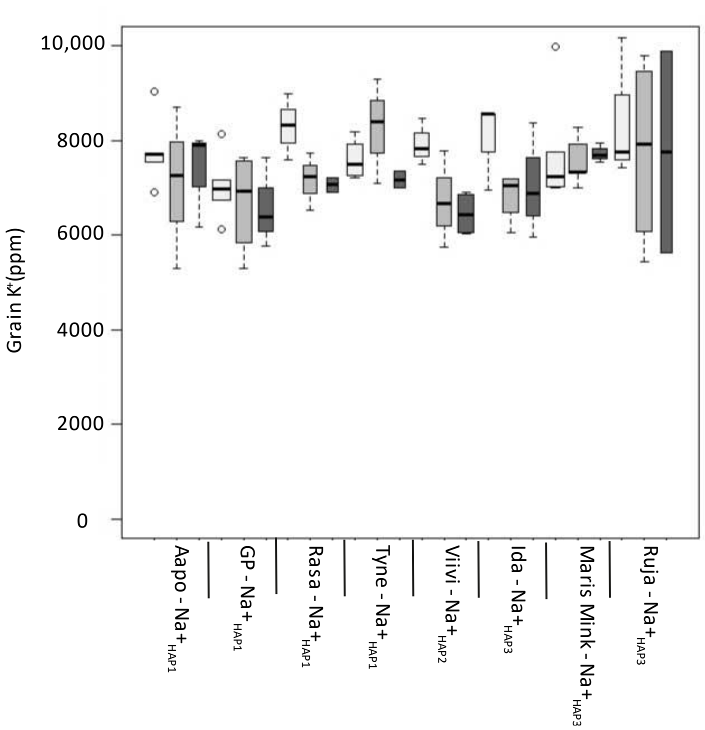

Supplementary Figure 4: Influence of L189P polymorphism in HvHKT1;5 on shoot Na^+ accumulation. Fifth leaf Na+ content after barley accessions were exposed to different levels of NaCl at the fourth leaf stage of development. For boxplots the horizontal bar of the boxplot shows the median, the box delineates the first and third quartile, and the whiskers show $+/-1.5 \times IQR$. White bars indicate 0mM of NaCl, light grey bars indicate 150mM NaCl, and dark grey indicates 250Mm NaCl added to plants.

Supplementary Figure 4

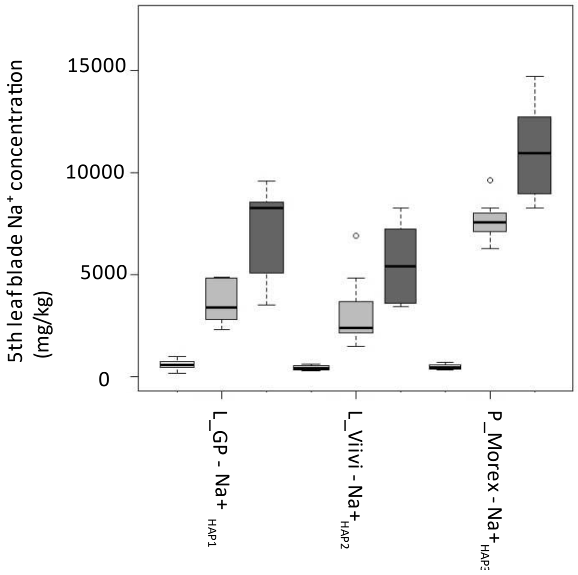

Supplementary Figure 5: Multiple alignment of species HKT orthologues

Supplementary Figure 5

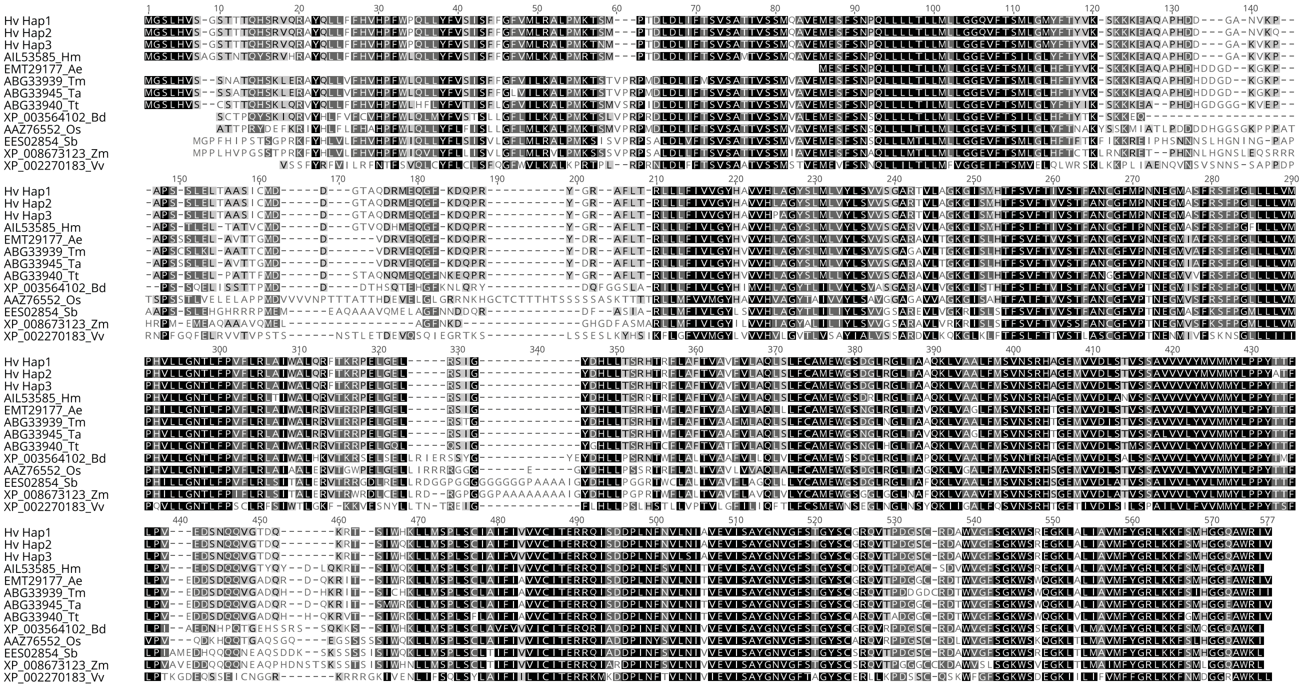

Supplementary Figure 6: Geographical distribution of L189P in barley germplasm.

a. Geographical distribution of L189P in *HvHKT1;5* in *H. spontaneum*. Location of accessions containing L189 in blue and 189P in red. **b**. Geographical distribution of L189P in *HvHKT1;5* in *H. vulgare landraces*. Location of accessions containing L189 in blue ($Na^{+}_{HAP1,}$ Na^{+}_{HAP2}) and 189P in red (Na^{+}_{HAP3}).

Supplementary Figure 6

A.

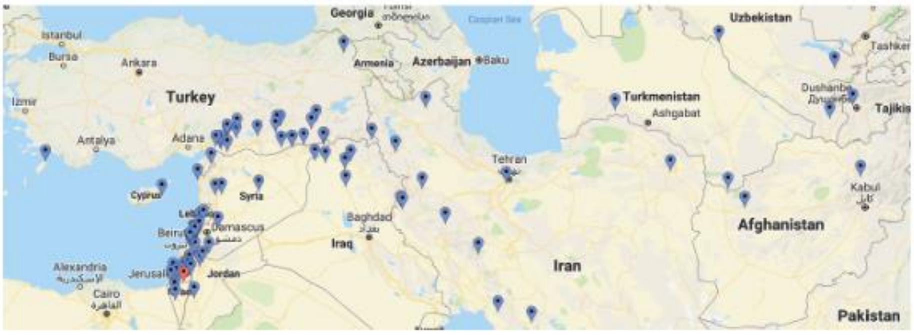

B.

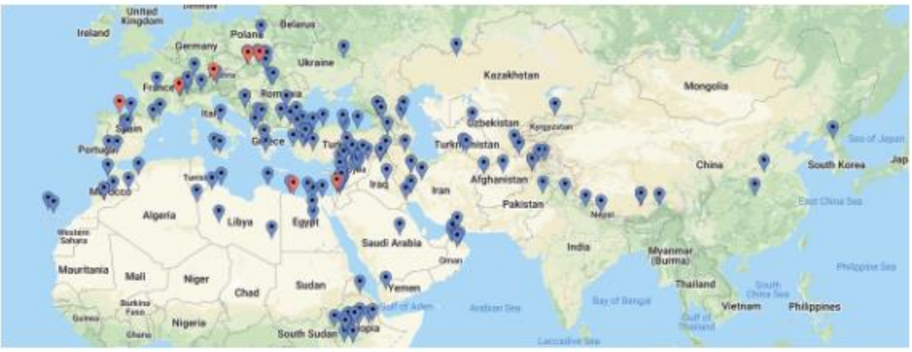

Supplementary Figure 7. Distribution of *HvHKT1;5* haplotypes in different genepools. **A.** Maximum likelihood tree of the coding sequence of *HvHKT1;5* in 73 *H. spontaneum*, including the 3 alleles of *HvHKT1;5*, Na^+_{HAP1}, Na^+_{HAP2} and Na^+_{HAP3}, identified in our analysis of elite 2-row spring barleys for reference. Colours highlight separate clades, red corresponding to the clade containing the accession representing the high sodium allele from the elite cultivars (Na^+_{HAP3}), dark blue the low grain sodium allele (Na^+_{HAP1}), and light blue representing the other low grain sodium allele from the elite cultivars (Na^+_{HAP2}). Branches representing the three haplotypes are indicated with their haplotype number and * represents FT064 which shares the same haplotype as HAP_3.
B. Maximum likelihood tree using 4000 SNPs selected randomly from across the genome of H. *spontaneum* landraces*,* accessions containing L189 are in blue and 189P in red. **C**. Maximum likelihood tree using 4000 SNPs selected randomly from across the genome of H. *vulgare landraces,* accessions containing L189 are in blue and 189P in red. **D.** Maximum likelihood tree using 4000 SNPs selected randomly from across the genome of *H. vulgare* cultivars*,* accessions containing L189 are in blue and 189P in red. Bootstrap values are included.

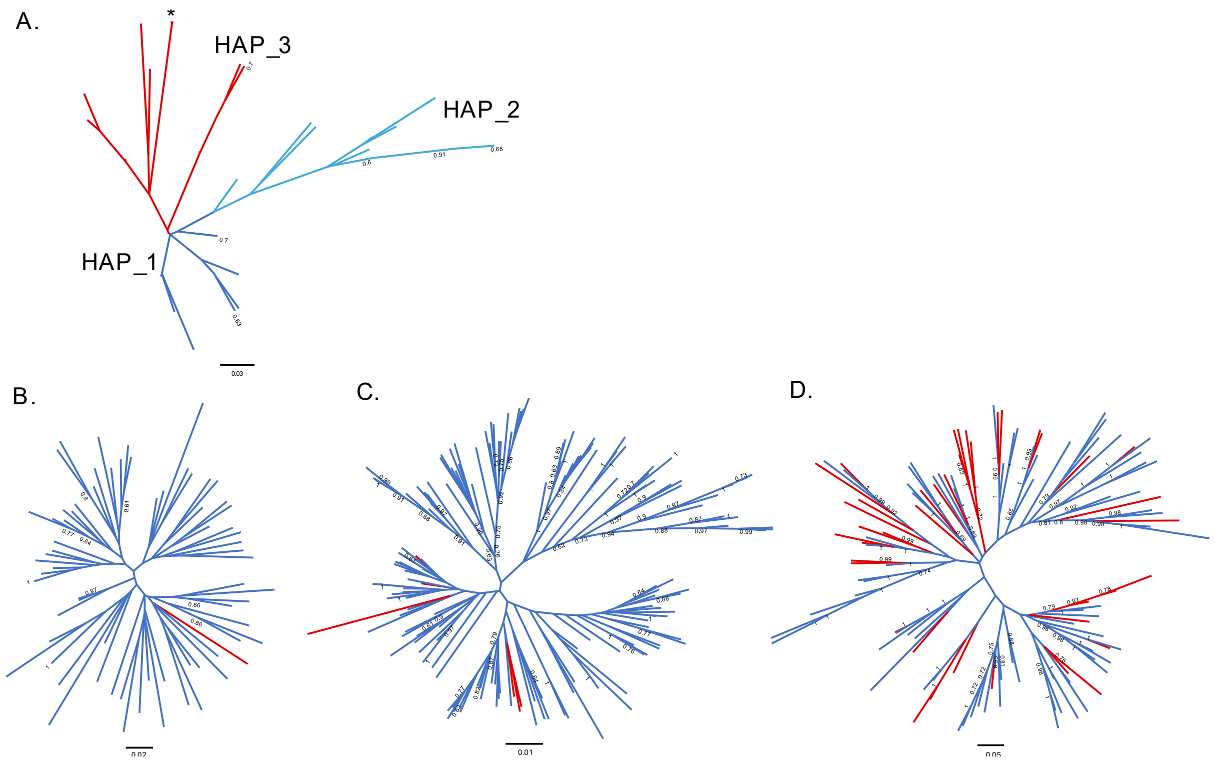

Supplementary Tables

Supplementary Table 1: Elite 2-row spring cultivars included in GWAS and sequenced for *HvHKT1:5*

Accession	Na23	K39	NCBI ref
AAPO	34.31	4796	MT312248
ACAPELLA	25.57	3464	MT312248
ADONIS	26.89	4535	MT312248
AGENDA	26.71	4691	MT312248
AKITA	29.41	3775	n.a
ALLIOT	41.76	4475	MT312248
AMOURETTE	27.96	4792	MT312248
ANACONDA	35.98	4560	MT312248
ANAIS	26.66	4397	MT312248
ANNABELL	20.9	4596	MT312248
APPALOOSA	70.94	4478	MT312250
ARDILA	24.59	4164	MT312248
ASTORIA	40.29	4676	MT312248
ATHENA	23.28	4324	MT312248
ATHOS	42.12	4362	MT312248
AVEC	16.07	3525	MT312248
BARKE	45.98	4742	MT312250
BARONESSE	48.2	4890	MT312248
BERWICK	33.66	4537	MT312248
BERYLLIUM	48.17	4553	MT312250
BLENHEIM	37.04	4498	MT312248
BRAEMAR	29.01	4493	n.a
BRAHMS	38.04	3349	MT312250
BRAZIL	35.44	4641	MT312250
CALICO	69.23	4430	MT312250
CAMPALA	46.13	4529	MT312250
CATALINA	43.9	4128	MT312250
CELLAR	17.58	3350	MT312248
CHARIOT	24.22	4164	MT312248
CHIEFTAN	33.04	4261	MT312248
CHIME	41.87	4375	MT312250
CLARITY	23.47	3938	MT312248
CLASS	23.37	4179	n.a
CLEOPATRA	21.22	4113	n.a
COLADA	36.07	4725	MT312248
COOPER	45.27	4167	MT312250
CROYDON	20.92	3963	MT312249
CRUSADER	47.65	4360	MT312250
DANUTA	24.26	4780	MT312248

Accession	Na23	K39	NCBI ref
DELIBES	40.74	4734	MT312250
DERKADO	23.56	3400	MT312248
DEW	28.53	4297	MT312248
DRAUGHT	21.83	4324	MT312248
DRUM	33.49	4644	MT312248
EXTRACT	49.11	4400	MT312250
FAIRYTALE	28.86	4750	MT312248
FELICIE	29.77	4131	MT312248
FOXTROT	26.93	4319	MT312248
FRANKLIN	37.77	4745	MT312248
GLOBAL	17.65	3341	MT312248
GOLDIE	17.71	3464	MT312248
GRANTA	54.29	4397	MT312250
GUNDEL	48.49	4454	MT312250
HART	48.09	4483	MT312250
HASSAN	29.11	4110	MT312248
HEATHER	50.34	4327	MT312250
HELMI	38.85	4609	MT312250
HERIS	20.12	4240	MT312248
HERON	32.24	4377	MT312248
HOPPER	26.23	4304	MT312248
HORIZON	27.84	4694	MT312248
HOST	21	4200	MT312248
IDA	59.15	4003	MT312250
INDOLA	19.11	3349	MT312248
ISABELLA	33.46	4657	MT312248
JIVE	25.79	4309	n.a
KEOPS	31.3	4606	MT312248
KLAXON	43.72	4615	MT312248
KRISTAPS	20.3	2957	MT312248
LINDEN	27.76	4327	MT312248
LITHIUM	52.07	4718	MT312250
LIVET	27.05	4292	MT312248
MACAW	51.53	4631	n.a
MADRAS	44.18	4504	MT312250
MARISMINK	83.79	4761	MT312250
MAYPOLE	69.63	4437	n.a
MELITTA	20.51	4095	MT312248
MIKADO	34.15	4142	MT312250
MINSTREL	30.59	4598	MT312248
NERUDA	32.84	4872	MT312248

Accession	Na23	K39	NCBI ref
NIMBUS	61.23	3487	MT312250
NOVELLO	22.42	4292	MT312248
OPTIC	16.62	4211	n.a
PENTHOUSE	27.74	4298	n.a
PEWTER	30.06	4445	MT312248
PITCHER	18.08	3971	MT312248
PONGO	47.51	4909	MT312250
PRIMERA	36.5	2693	MT312250
QUARTET	28.36	3922	MT312250
QUENCH	30.54	5089	MT312248
RAGTIME	24.45	4329	MT312248
RAINBOW	26.58	4584	MT312248
RAKAIA	43.05	4712	MT312248
RASA	20.15	3364	MT312248
RIVIERA	36.58	4264	MT312248
ROXANA	40.46	4404	MT312250
RUJA	46.97	4515	MT312250
RUMMY	20.48	4522	MT312248
SALKA	41.74	5142	MT312248
SALOON	72.64	5562	MT312250
SCANDIUM	68.29	4862	MT312250
SEBASTIAN	32.33	5175	MT312248
SIMBA	38.91	5398	MT312248
SKITTLE	20.44	4394	MT312248
SPIRAL	29.19	4606	MT312249
STARLIGHT	20.27	4373	MT312248
STATIC	24.64	4578	MT312248
SW_MACSENA	27.2	3560	MT312250
SW_STELLA	31.16	2459	MT312248
TABORA	43.95	4131	MT312250
TANKARD	22.91	4264	MT312248
TAPHOUSE	98.76	4803	MT312250
TARTAN	31.77	4512	MT312248
THISTLE	50.34	4758	MT312250
THRIFT	56.95	3963	MT312250
TOBY	47.06	3412	MT312248
TRINIDAD	78.77	4655	MT312250
TROON	31.91	4936	MT312248
TUCSON	30.82	4955	MT312248
TURNBERRY	65.93	4630	MT312250
TYNE	18.76	2643	MT312248

Accession	Na23	K39	NCBI ref
URSA	23.18	4236	n.a
VANKKURI	34.44	4508	MT312248
VELVET	26.61	4698	MT312248
VIIVI	16.4	4303	MT312249
VISKOSA	19.58	4956	MT312248
WAGGON	27.8	5043	MT312248
WEITOR	44.01	4466	MT312250
WICKET	68.25	4687	MT312250
WREN	43.05	3554	MT312250

Supplementary Table 2: Gene models in region identified on 4H as being significantly associated with grain Na+ content.

Gene Name	AGP Start	AGP End	Annotation
HORVU4Hr1G087780.1	638223190	638224749	Bifunctional inhibitor/lipid-transfer protein/seed storage 2S albumin superfamily protein
HORVU4Hr1G087790.2	638223460	638224442	undescribed protein
HORVU4Hr1G087800.1	638256143	638257008	Bifunctional inhibitor/lipid-transfer protein/seed storage 2S albumin superfamily protein
HORVU4Hr1G087810.1	638307473	638308181	undescribed protein
HORVU4Hr1G087820.1	638308517	638309341	undescribed protein
HORVU4Hr1G087830.1	638309936	638310439	unknown function
HORVU4Hr1G087840.3	638318732	638319925	Protein of unknown function (DUF1218)
HORVU4Hr1G087850.1	638333457	638333868	60S ribosomal protein L27-3
HORVU4Hr1G087860.1	638378121	638378871	Bifunctional inhibitor/lipid-transfer protein/seed storage 2S albumin superfamily protein
HORVU4Hr1G087870.1	638388268	638389146	Bifunctional inhibitor/lipid-transfer protein/seed storage 2S albumin superfamily protein
HORVU4Hr1G087880.1	638428432	638428842	60S ribosomal protein L27-3
HORVU4Hr1G087890.1	638478739	638479019	undescribed protein
HORVU4Hr1G087900.7	638479133	638484242	Flavin-containing monooxygenase family protein
HORVU4Hr1G087910.1	638486373	638488116	HXXXD-type acyl-transferase family protein
HORVU4Hr1G087920.1	638512331	638512750	Bifunctional inhibitor/lipid-transfer protein/seed storage 2S albumin superfamily protein
HORVU4Hr1G087930.1	638513260	638513517	undescribed protein
HORVU4Hr1G087940.1	638513647	638513841	unknown function
HORVU4Hr1G087950.1	638514999	638515151	Retrotransposon protein, putative, unclassified
HORVU4Hr1G087960.1	638634849	638636785	Sodium transporter HKT1
HORVU4Hr1G087970.1	638676269	638677708	TLD-domain containing nucleolar protein
HORVU4Hr1G087980.1	638774044	638774818	zinc finger protein 4
HORVU4Hr1G087990.6	639072081	639073349	Endoglucanase 15
HORVU4Hr1G088000.1	639080469	639080759	undescribed protein
HORVU4Hr1G088010.1	639196531	639196985	Protein TIME FOR COFFEE
HORVU4Hr1G088020.4	639208559	639213648	Protein NRT1/ PTR FAMILY 6.3

HORVU4Hr1G088030.1	639209570	639210052	undescribed protein
HORVU4Hr1G088040.1	639211354	639211737	undescribed protein
HORVU4Hr1G088050.1	639211900	639212080	undescribed protein
HORVU4Hr1G088060.4	639214025	639219029	Oxysterol-binding protein-related protein 2A
HORVU4Hr1G088070.1	639220179	639220679	undescribed protein
HORVU4Hr1G088080.4	639392933	639395123	expansin B2
HORVU4Hr1G088090.4	639495893	639498697	receptor kinase 2
HORVU4Hr1G088100.1	639682340	639682855	unknown function
HORVU4Hr1G088110.1	639706538	639707881	Eukaryotic aspartyl protease family protein
HORVU4Hr1G088120.1	639717292	639717831	Retrotransposon protein
HORVU4Hr1G088130.3	639721611	639722958	UvrB/uvrC motif-containing protein isoform 5
HORVU4Hr1G088140.2	639752757	639755359	expansin B2
HORVU4Hr1G088150.1	639767541	639768193	undescribed protein
HORVU4Hr1G088160.1	639772779	639774122	Eukaryotic aspartyl protease family protein
HORVU4Hr1G088170.1	639865436	639868763	Transposon protein, putative, CACTA, En/Spm sub-class
HORVU4Hr1G088180.1	639883596	639884246	undescribed protein
HORVU4Hr1G088190.9	639939694	639946933	2-phosphoglycerate kinase
HORVU4Hr1G088200.1	639942466	639943237	Endonuclease-reverse transcriptase HmRTE-e01
HORVU4Hr1G088220.1	639950849	639951108	undescribed protein
HORVU4Hr1G088210.35	639956048	639966348	CCR4-NOT transcription complex subunit 1
HORVU4Hr1G088230.1	640109804	640110976	Transposon protein, putative, CACTA, En/Spm sub-class
HORVU4Hr1G088240.1	640111176	640112560	unknown function
HORVU4Hr1G088250.15	640120326	640127451	undescribed protein
HORVU4Hr1G088260.1	640127727	640127928	undescribed protein
HORVU4Hr1G088270.14	640198187	640208474	ATP-dependent RNA helicase, putative
HORVU4Hr1G088280.2	640310745	640313017	GATA transcription factor 2
HORVU4Hr1G088290.1	640317992	640319088	undescribed protein
HORVU4Hr1G088300.1	640320298	640321148	undescribed protein

HORVU4Hr1G088310.1	640327918	640328758	undescribed protein
HORVU4Hr1G088320.1	640333038	640333841	Double Clp-N motif-containing P-loop nucleoside triphosphate hydrolases superfamily protein
HORVU4Hr1G088330.1	640393516	640393749	undescribed protein
HORVU4Hr1G088340.10	640421579	640432476	2-oxoglutarate (2OG) and Fe(II)-dependent oxygenase superfamily protein
HORVU4Hr1G088350.10	640434928	640441561	Agenet domain containing protein, expressed
HORVU4Hr1G088360.1	640445110	640445369	undescribed protein
HORVU4Hr1G088370.4	640445428	640449337	undescribed protein
HORVU4Hr1G088380.3	640445565	640449635	undescribed protein
HORVU4Hr1G088390.1	640491514	640497006	Coatomer, alpha subunit
HORVU4Hr1G088400.5	640497538	640499359	undescribed protein
HORVU4Hr1G088410.2	640498962	640499870	unknown function
HORVU4Hr1G088420.1	640500341	640500764	undescribed protein
HORVU4Hr1G088440.10	640503829	640510906	Methionyl-tRNA formyltransferase
HORVU4Hr1G088430.17	640504902	640510925	Plant protein of unknown function (DUF828) with plant pleckstrin homology-like region
HORVU4Hr1G088450.7	640515684	640518342	Plant protein of unknown function (DUF828) with plant pleckstrin homology-like region
HORVU4Hr1G088460.1	640520324	640520646	Serine/threonine-protein phosphatase 7 long form-like protein
HORVU4Hr1G088470.27	640535795	640538638	phosphatidylserine decarboxylase 1
HORVU4Hr1G088480.28	640540544	640546585	Chromosome 3B, genomic scaffold, cultivar Chinese Spring
HORVU4Hr1G088490.1	640547189	640550955	Cell number regulator 6
HORVU4Hr1G088500.2	640555331	640556013	undescribed protein
HORVU4Hr1G088510.1	640560265	640561373	undescribed protein
HORVU4Hr1G088520.1	640580304	640581107	Core-2/I-branching beta-1,6-N-acetylglucosaminyltransferase family protein
HORVU4Hr1G088530.1	640584186	640586647	Eukaryotic aspartyl protease family protein
HORVU4Hr1G088540.8	640595868	640611352	Chromodomain-helicase-DNA-binding protein 1-like
HORVU4Hr1G088550.1	640605004	640605309	undescribed protein
HORVU4Hr1G088570.2	640630455	640638112	Leucine-rich repeat receptor-like protein kinase family protein
HORVU4Hr1G088560.2	640630460	640640807	unknown function
HORVU4Hr1G088580.5	640641588	640647209	Leucine-rich repeat receptor-like protein kinase family protein

HORVU4Hr1G088590.6	640647353	640649638	unknown function
HORVU4Hr1G088600.4	640650819	640654398	Leucine-rich repeat receptor-like protein kinase family protein
HORVU4Hr1G088610.1	640662818	640665994	Quinone oxidoreductase
HORVU4Hr1G088620.1	640666350	640668581	Phosphoglycerate mutase family protein
HORVU4Hr1G088630.2	640671280	640671753	unknown function
HORVU4Hr1G088640.1	640673927	640674503	Retrotransposon protein, putative, unclassified
HORVU4Hr1G088650.6	640685421	640696260	protein kinase family protein
HORVU4Hr1G088660.1	640701625	640703561	E3 ubiquitin-protein ligase PRT6
HORVU4Hr1G088670.3	640706584	640707045	unknown function
HORVU4Hr1G088680.1	640707872	640709468	unknown function
HORVU4Hr1G088690.1	640711150	640712652	General transcription factor IIH subunit 5
HORVU4Hr1G088700.2	640716984	640726190	ARABIDILLO-1
HORVU4Hr1G088710.1	640725147	640726035	undescribed protein
HORVU4Hr1G088720.10	640726975	640730280	Palmitoyl-protein thioesterase 1
HORVU4Hr1G088730.1	640731610	640733054	undescribed protein
HORVU4Hr1G088740.1	640731612	640733092	Class I glutamine amidotransferase-like superfamily protein
HORVU4Hr1G088750.1	640899336	640899443	undescribed protein
HORVU4Hr1G088760.17	641042283	641047868	Long chain base biosynthesis protein 2d
HORVU4Hr1G088770.5	641043282	641046740	undescribed protein
HORVU4Hr1G088780.1	641086962	641088357	early nodulin-like protein 8
HORVU4Hr1G088790.5	641091033	641094510	arginine/serine-rich splicing factor 35
HORVU4Hr1G088800.1	641133970	641134732	undescribed protein
HORVU4Hr1G088810.1	641141491	641142102	Retrotransposon protein, putative, unclassified
HORVU4Hr1G088820.1	641145850	641152993	undescribed protein
HORVU4Hr1G088830.2	641145875	641152894	undescribed protein
HORVU4Hr1G088840.2	641153746	641156383	Post-GPI attachment to proteins factor 3
HORVU4Hr1G088850.4	641157517	641161403	Protein CHAPERONE-LIKE PROTEIN OF POR1, chloroplastic
HORVU4Hr1G088860.1	641185538	641189869	Major facilitator superfamily protein

HORVU4Hr1G088870.1	641191823	641192977	Mitochondrial import inner membrane translocase subunit TIM23-1
HORVU4Hr1G088880.7	641237832	641244215	kinesin 4
HORVU4Hr1G088890.2	641253106	641254865	Protein NRT1/ PTR FAMILY 8.1
HORVU4Hr1G088900.3	641255865	641265633	Alanine--tRNA ligase
HORVU4Hr1G088910.12	641271573	641284731	Protein kinase superfamily protein
HORVU4Hr1G088920.1	641290897	641291740	unknown function
HORVU4Hr1G088930.2	641293496	641295060	P-loop containing nucleoside triphosphate hydrolases superfamily protein
HORVU4Hr1G088940.1	641382061	641382844	undescribed protein
HORVU4Hr1G088950.1	641383076	641384750	undescribed protein
HORVU4Hr1G088960.2	641404116	641405408	undescribed protein
HORVU4Hr1G088970.1	641404825	641406494	undescribed protein
HORVU4Hr1G088980.1	641405536	641408090	undescribed protein
HORVU4Hr1G088990.1	641408194	641411623	undescribed protein
HORVU4Hr1G089000.1	641423398	641423880	Transposon protein, putative, Pong sub-class
HORVU4Hr1G089010.1	641429665	641431292	undescribed protein
HORVU4Hr1G089020.1	641454180	641454425	Retrotransposon protein, putative, unclassified, expressed
HORVU4Hr1G089030.1	641464335	641465907	Protein of unknown function, DUF584
HORVU4Hr1G089040.1	641468548	641468670	undescribed protein
HORVU4Hr1G089050.1	641468869	641469015	unknown function
HORVU4Hr1G089060.3	641590688	641595759	L-gulonolactone oxidase 5
HORVU4Hr1G089070.1	641592081	641592694	undescribed protein
HORVU4Hr1G089080.3	641600093	641606961	UDP-N-acetylglucosamine--peptide N-acetylglucosaminyltransferase 110 kDa subunit
HORVU4Hr1G089090.2	641608354	641613003	Chaperone protein DnaK
HORVU4Hr1G089100.1	641617531	641618461	unknown function
HORVU4Hr1G089110.1	641759666	641760070	undescribed protein
HORVU4Hr1G089120.1	641771191	641775630	Trihelix transcription factor GT-2
HORVU4Hr1G089130.1	641786871	641790804	TTF-type zinc finger protein with HAT dimerisation domain
HORVU4Hr1G089140.1	641790981	641791310	hAT dimerisation domain-containing protein / transposase-related

HORVU4Hr1G089150.1	641794439	641795275	undescribed protein
HORVU4Hr1G089160.1	641798627	641801315	unknown function
HORVU4Hr1G089170.1	641806339	641807327	undescribed protein
HORVU4Hr1G089180.1	641837276	641838618	Retrotransposon protein, putative, unclassified
HORVU4Hr1G089190.1	641839374	641840867	undescribed protein
HORVU4Hr1G089200.2	641851862	641853556	Protein kinase superfamily protein
HORVU4Hr1G089210.1	641904210	641906080	Plant protein of unknown function (DUF247)
HORVU4Hr1G089220.2	641910099	641911620	Protein LURP-one-related 8
HORVU4Hr1G089230.1	641924251	641926641	Cytochrome P450 superfamily protein
HORVU4Hr1G089240.1	642024767	642025697	unknown function
HORVU4Hr1G089250.1	642027381	642027828	Retrotransposon protein, putative, unclassified
HORVU4Hr1G089260.1	642028955	642029657	undescribed protein
HORVU4Hr1G089270.1	642064582	642066848	Zinc finger CCCH domain-containing protein 20
HORVU4Hr1G089280.5	642067124	642071227	Transmembrane and coiled-coil domain-containing protein 4
HORVU4Hr1G089300.1	642149539	642149808	undescribed protein
HORVU4Hr1G089310.19	642155943	642276817	AP-2 complex subunit alpha-1
HORVU4Hr1G089310.19	642155943	642276817	AP-2 complex subunit alpha-1
HORVU4Hr1G089320.1	642174952	642176162	Zinc finger CCCH domain-containing protein 44
HORVU4Hr1G089330.2	642199303	642201938	2-oxoglutarate (2OG) and Fe(II)-dependent oxygenase superfamily protein
HORVU4Hr1G089340.1	642200689	642201204	undescribed protein
HORVU4Hr1G089350.4	642210402	642217528	WD repeat-containing protein 6
HORVU4Hr1G089360.1	642223610	642225409	Flavin-containing monooxygenase family protein
HORVU4Hr1G089370.1	642233366	642234607	General transcription factor 2-related zinc finger protein
HORVU4Hr1G089380.1	642236484	642236702	unknown function
HORVU4Hr1G089390.1	642244367	642244741	unknown function
HORVU4Hr1G089400.3	642249025	642249328	L-tyrosine decarboxylase
HORVU4Hr1G089410.1	642261253	642262994	2-oxoglutarate (2OG) and Fe(II)-dependent oxygenase superfamily protein
HORVU4Hr1G089290.7	642309174	642316391	Zinc finger CCCH domain-containing protein 19

HORVU4Hr1G089420.1	642383189	642383376	unknown function
HORVU4Hr1G089430.1	642393080	642393434	undescribed protein
HORVU4Hr1G089440.1	642462273	642462618	Retrotransposon protein, putative, unclassified
HORVU4Hr1G089450.1	642496560	642498144	NAC domain protein,
HORVU4Hr1G089460.1	642513360	642523797	receptor-like protein kinase 1
HORVU4Hr1G089470.2	642527742	642530387	dihydroflavonol 4-reductase
HORVU4Hr1G089480.1	642535429	642537069	2-oxoglutarate (2OG) and Fe(II)-dependent oxygenase superfamily protein
HORVU4Hr1G089490.2	642543999	642544800	Non-specific lipid-transfer protein 2G
HORVU4Hr1G089500.1	642559204	642559830	Non-specific lipid-transfer protein 2G
HORVU4Hr1G089510.3	642560707	642564722	beta-amylase 5
HORVU4Hr1G089520.1	642568886	642570443	Heavy metal-associated domain, putative
HORVU4Hr1G089530.1	642569427	642569815	undescribed protein
HORVU4Hr1G089540.2	642587402	642590164	Remorin family protein
HORVU4Hr1G089550.1	642984437	642985124	undescribed protein
HORVU4Hr1G089560.2	643004384	643006737	Chromosome 3B, genomic scaffold, cultivar Chinese Spring
HORVU4Hr1G089570.1	643009098	643009715	undescribed protein
HORVU4Hr1G089580.1	643012334	643013799	sulfotransferase 4B
HORVU4Hr1G089600.1	643055182	643056109	undescribed protein
HORVU4Hr1G089590.18	643060225	643064076	Small nuclear ribonucleoprotein family protein
HORVU4Hr1G089610.2	643065153	643069109	glycerol-3-phosphate acyltransferase 3
HORVU4Hr1G089620.1	643184706	643185401	Folylpolyglutamate synthase
HORVU4Hr1G089630.1	643221126	643222775	undescribed protein
HORVU4Hr1G089640.1	643365019	643366386	undescribed protein
HORVU4Hr1G089650.1	643371175	643371873	Retrotransposon protein, putative, Ty1-copia subclass
HORVU4Hr1G089660.1	643378290	643379142	NAC domain containing protein 32
HORVU4Hr1G089670.1	643380058	643381950	Galactosyl transferase GMA12/MNN10 family protein
HORVU4Hr1G089680.1	643396960	643397346	undescribed protein
HORVU4Hr1G089690.3	643401272	643403295	FLOWERING LOCUS T protein, putative

HORVU4Hr1G089700.1	643406292	643406675	unknown function
HORVU4Hr1G089710.1	643432434	643433091	Chromosome 3B, genomic scaffold, cultivar Chinese Spring
HORVU4Hr1G089720.1	643438068	643441387	polyubiquitin 10
HORVU4Hr1G089730.5	643447233	643450680	DNA (cytosine-5)-methyltransferase DRM2
HORVU4Hr1G089740.1	643469531	643471438	unknown function
HORVU4Hr1G089750.1	643496721	643497628	nicotianamine synthase 3
HORVU4Hr1G089750.1	643496721	643497628	nicotianamine synthase 3
HORVU4Hr1G089760.1	643505736	643506752	rRNA N-glycosidase
HORVU4Hr1G089770.1	643510944	643513556	unknown function
HORVU4Hr1G089780.1	643514792	643515901	rRNA N-glycosidase
HORVU4Hr1G089790.1	643531658	643532317	unknown function
HORVU4Hr1G089800.1	643539316	643553308	undescribed protein
HORVU4Hr1G089810.1	643554452	643554697	undescribed protein
HORVU4Hr1G089820.7	643555408	643563271	THO complex subunit 1
HORVU4Hr1G089830.2	643624525	643625498	unknown function
HORVU4Hr1G089840.1	643628611	643629033	unknown function
HORVU4Hr1G089850.2	643641394	643644339	rRNA N-glycosidase
HORVU4Hr1G089860.1	643645935	643646389	unknown function
HORVU4Hr1G089870.1	643649672	643650337	nicotianamine synthase 4
HORVU4Hr1G089880.1	643667668	643670890	Embryogenesis transmembrane protein-like
HORVU4Hr1G089890.1	643676698	643680809	Embryogenesis transmembrane protein-like
HORVU4Hr1G089900.1	643698720	643699836	HXXXD-type acyl-transferase family protein
HORVU4Hr1G089910.8	643754944	643757160	Peptidoglycan-binding LysM domain-containing protein
HORVU4Hr1G089920.1	643756201	643756656	undescribed protein
HORVU4Hr1G089930.1	643802641	643803933	Dirigent, putative, expressed
HORVU4Hr1G089940.1	643849687	643850352	undescribed protein
HORVU4Hr1G089950.2	644014518	644014748	Beta-fructofuranosidase, soluble isoenzyme I
HORVU4Hr1G089960.1	644025240	644025680	unknown function

HORVU4Hr1G089970.1	644027463	644027603	HAT family dimerisation domain containing protein, expressed
HORVU4Hr1G089980.1	644027616	644029495	TTF-type zinc finger protein with HAT dimerisation domain
HORVU4Hr1G089990.4	644043699	644046293	Protein WEAK CHLOROPLAST MOVEMENT UNDER BLUE LIGHT 1
HORVU4Hr1G090000.4	644046935	644048843	unknown protein; BEST Arabidopsis thaliana protein match is: unknown protein .
HORVU4Hr1G090010.1	644327130	644327811	undescribed protein
HORVU4Hr1G090020.2	644335686	644338588	Leucine-rich repeat receptor-like protein kinase family protein
HORVU4Hr1G090030.3	644342602	644351407	Homeobox-leucine zipper protein HOX10
HORVU4Hr1G090040.1	644352889	644353678	unknown function
HORVU4Hr1G090050.3	644380174	644381317	Peroxidase superfamily protein
HORVU4Hr1G090060.1	644390902	644391988	unknown function
HORVU4Hr1G090070.1	644399260	644399972	undescribed protein
HORVU4Hr1G090080.1	644409589	644417798	Malate dehydrogenase
HORVU4Hr1G090090.4	644434690	644435863	Heat stress transcription factor C-2a
HORVU4Hr1G090100.1	644442321	644443454	undescribed protein
HORVU4Hr1G090110.5	644449280	644450579	Nascent polypeptide-associated complex subunit beta
HORVU4Hr1G090120.1	644475545	644476628	germin-like protein 9
HORVU4Hr1G090130.1	644480922	644481931	germin-like protein 2
HORVU4Hr1G090140.1	644484458	644485142	germin-like protein 9
HORVU4Hr1G090150.1	644506183	644508490	peptidyl-prolyl cis-trans isomerases
HORVU4Hr1G090160.1	644508965	644513417	DNA repair endonuclease UVH1
HORVU4Hr1G090180.1	644543466	644543732	undescribed protein
HORVU4Hr1G090190.4	644547127	644548791	germin-like protein 2
HORVU4Hr1G090170.17	644551948	644565388	tRNA/rRNA methyltransferase (SpoU) family protein
HORVU4Hr1G090200.1	644566417	644566659	undescribed protein
HORVU4Hr1G090210.1	644566855	644571558	DNA repair endonuclease UVH1
HORVU4Hr1G090220.1	644572117	644574424	Peptidyl-prolyl cis-trans isomerase-like 3

Supplementary Table 3: *H. spontaneum* and *H. vulgare landrace* HvHKT1;5 genotypic data. Includes all non-synonymous SNPs for *H spontaneum* accessions, location in amino acid sequence is provides in top header row, and location in CDS in second header row.

Germplasm	L189P	NCBI ref	Q18H	F26S	H29R	S56N	E102Q	K130N	P133Q	R166H	L189P	V223I	G285C	V308M	V310G
			G54T	T77C	A86G	G170A	G304C	A393T	C401A	G498A	T567C	G670A	G854T	G923A	T920G
H. spontaneum	P	MT334696	G	T	A	A	G	A	C	G	C	A	G	G	T
H. spontaneum	L	MT334697	.	C	.	G	C	.	.	.	T	.	.	.	.
H. spontaneum	L	MT334698	.	C	.	.	.	.	.	.	T	.	.	.	.
H. spontaneum	L	MT334699	.	C	.	G	C	.	.	.	T	.	.	.	.
H. spontaneum	L	MT334700	.	C	.	G	C	.	.	.	T	.	.	.	.
H. spontaneum	L	MT334701	.	C	.	G	C	.	.	.	T	.	.	.	.
H. spontaneum	L	MT334702	.	C	.	G	C	.	.	.	T	.	.	.	.
H. spontaneum	L	MT334703	.	C	.	G	C	.	.	.	T	.	.	.	.
H. spontaneum	L	MT334704	.	C	.	G	C	.	.	.	T	.	.	.	.
H. spontaneum	L	MT334705	.	C	.	G	C	.	.	.	T	.	.	.	.
H. spontaneum	L	MT334706	.	C	.	G	C	.	.	.	T	.	.	.	.
H. spontaneum	L	MT334707	.	C	.	G	C	.	.	.	T	.	.	.	.
H. spontaneum	L	MT334708	.	C	.	G	C	.	.	.	T	.	.	.	.
H. spontaneum	L	MT334709	.	C	.	G	C	.	.	.	T	.	.	.	.
H. spontaneum	L	MT334710	.	C	.	G	C	.	.	.	T	.	.	.	.
H. spontaneum	L	MT334711	.	C	.	G	C	.	.	.	T	.	.	.	.
H. spontaneum	L	MT334712	.	C	.	G	C	.	.	.	T	.	.	.	.
H. spontaneum	L	MT334713	.	C	.	G	C	.	.	.	T	.	.	.	.
H. spontaneum	L	MT334714	.	C	.	G	C	.	.	.	T	.	.	.	.
H. spontaneum	L	MT334715	.	C	.	G	C	.	.	.	T	.	.	.	.
H. spontaneum	L	MT334716	.	C	.	G	C	.	.	.	T	.	.	.	.
H. spontaneum	L	MT334717	.	C	.	G	C	.	.	.	T	.	.	.	.
H. spontaneum	L	MT334718	.	C	.	G	C	.	.	.	T	.	.	.	.
H. spontaneum	L	MT334719	.	C	.	G	C	.	.	.	T	.	.	.	.
H. spontaneum	L	MT334720	.	C	.	G	C	.	.	.	T	.	.	.	.

H. spontaneum	L	MT334721	.	C	.	G	C	.	.	.	T	.	.	.	.

H. spontaneum	L	MT334722	.	C	.	G	C	.	.	.	T	.	.	.	.
H. spontaneum	L	MT334723	.	C	.	G	C	.	.	.	T	.	.	.	.
H. spontaneum	L	MT334724	.	C	.	G	C	.	.	.	T	G	.	.	.
H. spontaneum	L	MT334725	.	C	.	G	C	T	.	.	T	G	.	.	.
H. spontaneum	L	MT334726	.	C	.	G	C	T	.	.	T	G	.	.	.
H. spontaneum	L	MT334727	.	C	.	G	C	T	.	.	T	G	.	.	.
H. spontaneum	L	MT334728	.	C	.	G	C	T	.	.	T	G	T	.	.
H. spontaneum	L	MT334729	.	C	.	G	C	T	.	.	T	G	T	.	.
H. spontaneum	L	MT334730	.	C	.	G	.	.	.	.	T	.	.	.	G
H. spontaneum	L	MT334731	.	C	.	G	.	.	.	.	T	.	.	.	.
H. spontaneum	L	MT334732	.	C	.	G	.	.	.	.	T	.	.	.	.
H. spontaneum	L	MT334733	.	C	.	G	.	.	.	.	T	.	.	.	.
H. spontaneum	L	MT334734	.	C	.	G	.	.	.	.	T	.	.	.	.
H. spontaneum	L	MT334735	.	C	.	G	.	.	.	.	T	.	.	.	.
H. spontaneum	L	MT334736	.	C	.	G	.	.	.	.	T	.	.	.	.

Germplasm	L189P	NCBI ref	Q18H	F26S	H29R	S56N	E102Q	K130N	P133Q	R166H	L189P	V223I	G285C	V308M	V310G
			G54T	T77C	A86G	G170A	G304C	A393T	C401A	G498A	T567C	G670A	G854T	G923A	T920G
H. spontaneum	L	MT334737	.	C	.	G	.	.	.	.	T	.	.	.	.
H. spontaneum	L	MT334738	.	C	.	G	.	.	.	.	T	.	.	.	.
H. spontaneum	L	MT334739	.	C	.	G	.	.	.	.	T	.	.	.	.
H. spontaneum	L	MT334740	.	C	.	G	.	.	.	.	T	.	.	A	.
H. spontaneum	L	MT334741	.	C	.	G	.	.	.	.	T	.	.	A	.
H. spontaneum	L	MT334742	.	C	.	G	.	.	.	.	T	.	.	.	.
H. spontaneum	L	MT334743	.	C	.	G	.	.	.	.	T	.	.	.	.
H. spontaneum	L	MT334744	.	C	.	G	.	.	.	.	T	.	.	.	.
H. spontaneum	L	MT334745	.	C	.	G	.	.	.	.	T	.	.	.	.
H. spontaneum	L	MT334746	.	C	.	G	.	.	.	.	T	.	.	.	.
H. spontaneum	L	MT334747	.	C	.	G	.	.	.	.	T	.	.	.	.

H. spontaneum	L	MT334748	.	C	.	G	.	.	.	.	T	.	.	.	.
H. spontaneum	L	MT334749	.	C	.	G	.	.	.	.	T	.	.	.	.
H. spontaneum	L	MT334750	.	C	.	G	.	.	.	.	T	.	.	.	.
H. spontaneum	L	MT334751	.	C	.	G	.	.	.	.	T	.	.	.	.
H. spontaneum	L	MT334752	.	C	.	G	.	.	.	.	T	.	.	.	.
H. spontaneum	L	MT334753	.	C	.	G	.	.	.	.	T	.	.	.	.
H. spontaneum	L	MT334754	.	C	.	G	.	.	.	.	T	.	.	.	.
H. spontaneum	L	MT334755	.	C	.	G	.	.	.	.	T	.	.	.	.
H. spontaneum	L	MT334756	.	C	.	G	.	.	.	.	T	.	.	.	.
H. spontaneum	L	MT334757	.	C	.	G	.	.	.	.	T	.	.	.	.
H. spontaneum	L	MT334758	.	C	.	G	.	.	.	.	T	.	.	.	.
H. spontaneum	L	MT334759	.	C	.	G	.	.	.	.	T	.	.	.	.
H. spontaneum	L	MT334760	.	C	.	G	.	.	.	.	T	.	.	.	.
H. spontaneum	L	MT334761	.	C	.	G	.	.	.	.	T	.	.	.	.
H. spontaneum	L	MT334762	.	C	.	G	.	.	.	.	T	.	.	.	.
H. spontaneum	L	MT334763	.	C	.	G	.	.	.	.	T	.	.	.	.
H. spontaneum	L	MT334764	.	C	.	G	.	.	.	.	T	.	.	.	.
H. spontaneum	L	MT334765	.	C	.	G	.	.	.	.	T	.	.	.	.
H. spontaneum	L	MT334766	.	C	.	G	.	.	.	.	T	.	.	.	.
H. spontaneum	L	MT334767	.	C	.	G	.	.	.	.	T	.	.	.	.
H. spontaneum	L	MT334768	.	C	.	G	.	T	.	A	T	G	.	.	.
H. spontaneum	L	MT334769	.	C	.	G	.	T	A	.	T	.	.	.	.
H. spontaneum	L	MT334770	.	C	.	G	.	T	A	.	T	G	.	.	.
H. spontaneum	L	MT334771	.	C	.	G	.	.	.	.	T	.	.	.	.
H. spontaneum	L	MT334772	.	C	.	G	.	.	.	.	T	.	.	.	.
H. spontaneum	L	MT334773	.	C	G	G	.	.	.	.	T	.	.	.	.
H. spontaneum	L	MT334774	.	C	G	G	.	.	.	.	T	.	.	.	.
H. spontaneum	L	MT334775	T	C	.	G	.	.	.	.	T	.	.	.	.

Germplasm	L189P	NCBI ref
H. vulgare landrace	P	n/a
H. vulgare landrace	P	n/a
H. vulgare landrace	P	n/a
H. vulgare landrace	P	n/a
H. vulgare landrace	P	n/a
H. vulgare landrace	P	n/a
H. vulgare landrace	P	n/a
H. vulgare landrace	L	n/a
H. vulgare landrace	L	n/a
H. vulgare landrace	L	n/a
H. vulgare landrace	L	n/a
H. vulgare landrace	L	n/a
H. vulgare landrace	L	n/a
H. vulgare landrace	L	n/a
H. vulgare landrace	L	n/a
H. vulgare landrace	L	n/a
H. vulgare landrace	L	n/a
H. vulgare landrace	L	n/a
H. vulgare landrace	L	n/a
H. vulgare landrace	L	n/a
H. vulgare landrace	L	n/a
H. vulgare landrace	L	n/a
H. vulgare landrace	L	n/a

H. vulgare landrace	L	n/a

H. vulgare landrace	L	n/a
H. vulgare landrace	L	n/a
H. vulgare landrace	L	n/a
H. vulgare landrace	L	n/a
H. vulgare landrace	L	n/a
H. vulgare landrace	L	n/a
H. vulgare landrace	L	n/a
H. vulgare landrace	L	n/a
H. vulgare landrace	L	n/a
H. vulgare landrace	L	n/a
H. vulgare landrace	L	n/a
H. vulgare landrace	L	n/a
H. vulgare landrace	L	n/a
H. vulgare landrace	L	n/a
H. vulgare landrace	L	n/a
H. vulgare landrace	L	n/a
H. vulgare landrace	L	n/a

Germplasm	L189P	NCBI ref
H. vulgare landrace	L	n/a
H. vulgare landrace	L	n/a
H. vulgare landrace	L	n/a
H. vulgare landrace	L	n/a
H. vulgare landrace	L	n/a
H. vulgare landrace	L	n/a
H. vulgare landrace	L	n/a
H. vulgare landrace	L	n/a
H. vulgare landrace	L	n/a

H. vulgare landrace	L	n/a
H. vulgare landrace	L	n/a
H. vulgare landrace	L	n/a
H. vulgare landrace	L	n/a
H. vulgare landrace	L	n/a
H. vulgare landrace	L	n/a
H. vulgare landrace	L	n/a
H. vulgare landrace	L	n/a
H. vulgare landrace	L	n/a
H. vulgare landrace	L	n/a
H. vulgare landrace	L	n/a
H. vulgare landrace	L	n/a
H. vulgare landrace	L	n/a
H. vulgare landrace	L	n/a
H. vulgare landrace	L	n/a
H. vulgare landrace	L	n/a
H. vulgare landrace	L	n/a
H. vulgare landrace	L	n/a
H. vulgare landrace	L	n/a
H. vulgare landrace	L	n/a
H. vulgare landrace	L	n/a
H. vulgare landrace	L	n/a
H. vulgare landrace	L	n/a
H. vulgare landrace	L	n/a
H. vulgare landrace	L	n/a
H. vulgare landrace	L	n/a
H. vulgare landrace	L	n/a
H. vulgare landrace	L	n/a

Germplasm	L189P	NCBI ref
H. vulgare landrace	L	n/a
H. vulgare landrace	L	n/a
H. vulgare landrace	L	n/a
H. vulgare landrace	L	n/a
H. vulgare landrace	L	n/a
H. vulgare landrace	L	n/a
H. vulgare landrace	L	n/a
H. vulgare landrace	L	n/a
H. vulgare landrace	L	n/a
H. vulgare landrace	L	n/a
H. vulgare landrace	L	n/a
H. vulgare landrace	L	n/a
H. vulgare landrace	L	n/a
H. vulgare landrace	L	n/a
H. vulgare landrace	L	n/a
H. vulgare landrace	L	n/a
H. vulgare landrace	L	n/a
H. vulgare landrace	L	n/a
H. vulgare landrace	L	n/a
H. vulgare landrace	L	n/a
H. vulgare landrace	L	n/a
H. vulgare landrace	L	n/a
H. vulgare landrace	L	n/a
H. vulgare landrace	L	n/a
H. vulgare landrace	L	n/a
H. vulgare landrace	L	n/a

H. vulgare landrace	L	n/a
H. vulgare landrace	L	n/a
H. vulgare landrace	L	n/a
H. vulgare landrace	L	n/a
H. vulgare landrace	L	n/a
H. vulgare landrace	L	n/a
H. vulgare landrace	L	n/a
H. vulgare landrace	L	n/a
H. vulgare landrace	L	n/a
H. vulgare landrace	L	n/a
H. vulgare landrace	L	n/a
H. vulgare landrace	L	n/a
H. vulgare landrace	L	n/a
H. vulgare landrace	L	n/a
H. vulgare landrace	L	n/a
H. vulgare landrace	L	n/a
H. vulgare landrace	L	n/a
H. vulgare landrace	L	n/a
H. vulgare landrace	L	n/a

Germplasm	L189P	NCBI ref
H. vulgare landrace	L	n/a
H. vulgare landrace	L	n/a
H. vulgare landrace	L	n/a
H. vulgare landrace	L	n/a
H. vulgare landrace	L	n/a
H. vulgare landrace	L	n/a
H. vulgare landrace	L	n/a

H. vulgare landrace	L	n/a
H. vulgare landrace	L	n/a
H. vulgare landrace	L	n/a
H. vulgare landrace	L	n/a
H. vulgare landrace	L	n/a
H. vulgare landrace	L	n/a
H. vulgare landrace	L	n/a
H. vulgare landrace	L	n/a
H. vulgare landrace	L	n/a
H. vulgare landrace	L	n/a
H. vulgare landrace	L	n/a
H. vulgare landrace	L	n/a
H. vulgare landrace	L	n/a
H. vulgare landrace	L	n/a
H. vulgare landrace	L	n/a
H. vulgare landrace	L	n/a
H. vulgare landrace	L	n/a
H. vulgare landrace	L	n/a
H. vulgare landrace	L	n/a
H. vulgare landrace	L	n/a
H. vulgare landrace	L	n/a
H. vulgare landrace	L	n/a
H. vulgare landrace	L	n/a
H. vulgare landrace	L	n/a
H. vulgare landrace	L	n/a
H. vulgare landrace	L	n/a
H. vulgare landrace	L	n/a
H. vulgare landrace	L	n/a

H. vulgare landrace	L	n/a
H. vulgare landrace	L	n/a
H. vulgare landrace	L	n/a
H. vulgare landrace	L	n/a
H. vulgare landrace	L	n/a
H. vulgare landrace	L	n/a

Germplasm	L189P	NCBI ref
H. vulgare landrace	L	n/a
H. vulgare landrace	L	n/a
H. vulgare landrace	L	n/a
H. vulgare landrace	L	n/a
H. vulgare landrace	L	n/a
H. vulgare landrace	L	n/a
H. vulgare landrace	L	n/a
H. vulgare landrace	L	n/a
H. vulgare landrace	L	n/a
H. vulgare landrace	L	n/a
H. vulgare landrace	L	n/a
H. vulgare landrace	L	n/a
H. vulgare landrace	L	n/a
H. vulgare landrace	L	n/a
H. vulgare landrace	L	n/a
H. vulgare landrace	L	n/a
H. vulgare landrace	L	n/a
H. vulgare landrace	L	n/a
H. vulgare landrace	L	n/a
H. vulgare landrace	L	n/a

Germplasm	L189P	NCBI ref	A379T	L382Q	Q402H	K403M	I416V	V436I	N438S	T463A	R481M	Q482E	V490L
			G1136A	T1145A	G1206C	A1208T	A1248G	G1306A	A1404G	A1384G	A1439T	G1441C	G1465C
H. spontaneum	P	MT334696	A	T	C	A	G	G	G	A	G	G	G
H. spontaneum	L	MT334697	.	.	.	.	A	.	A	G	.	.	.
H. spontaneum	L	MT334698	.	.	G	.	A	.	A	G	.	.	.
H. spontaneum	L	MT334699	.	A	.	.	A	.	.	G	.	.	C
H. spontaneum	L	MT334700	.	A	.	.	A	.	A	G	.	.	.
H. spontaneum	L	MT334701	.	A	.	.	A	.	A	G	.	.	.
H. spontaneum	L	MT334702	.	A	.	.	A	.	A	G	.	.	.
H. spontaneum	L	MT334703	.	A	.	.	A	.	A	G	.	.	.
H. spontaneum	L	MT334704	.	.	.	T	A	.	.	G	.	.	.
H. spontaneum	L	MT334705	.	.	.	T	A	.	.	G	.	.	.
H. spontaneum	L	MT334706	.	.	.	T	A	.	.	G	.	.	.
H. spontaneum	L	MT334707	.	.	.	T	A	.	.	G	.	.	.
H. spontaneum	L	MT334708	.	.	.	.	A	.	.	G	.	.	.
H. spontaneum	L	MT334709	.	.	.	.	A	A	.	G	.	.	.
H. spontaneum	L	MT334710	.	.	.	.	A	.	A	G	.	.	.
H. spontaneum	L	MT334711	.	.	.	.	A	.	A	G	.	.	.
H. spontaneum	L	MT334712	.	.	.	.	A	.	A	G	.	.	.
H. spontaneum	L	MT334713	.	.	.	.	A	.	A	G	.	.	.
H. spontaneum	L	MT334714	.	.	.	.	A	.	A	G	.	.	.
H. spontaneum	L	MT334715	.	.	.	.	A	.	A	G	.	.	.
H. spontaneum	L	MT334716	.	.	.	.	A	.	A	G	.	.	.
H. spontaneum	L	MT334717	.	.	.	.	A	.	A	G	.	.	.
H. spontaneum	L	MT334718	.	.	.	.	A	.	A	G	.	.	.
H. spontaneum	L	MT334719	.	.	.	.	A	.	A	G	.	.	.
H. spontaneum	L	MT334720	.	.	.	.	A	.	A	G	.	.	.
H. spontaneum	L	MT334721	.	.	.	.	A	.	A	G	.	.	.

H. spontaneum	L	MT334722	.	.	.	.	A	.	A	G	.	.	.
H. spontaneum	L	MT334723	.	.	.	.	A	.	A	G	.	.	.
H. spontaneum	L	MT334724	.	.	.	.	A	.	A	G	.	.	.
H. spontaneum	L	MT334725	G	.	G	.	A	.	A	G	.	.	.
H. spontaneum	L	MT334726	G	.	G	.	A	.	A	G	.	.	.
H. spontaneum	L	MT334727	G	.	G	.	A	.	A	G	.	.	.
H. spontaneum	L	MT334728	G	.	G	.	A	.	A	G	.	.	.
H. spontaneum	L	MT334729	G	.	G	.	A	.	A	G	.	.	.
H. spontaneum	L	MT334730	.	A	.	.	A	.	A	G	.	.	.
H. spontaneum	L	MT334731	.	A	.	.	A	.	A	G	.	.	.
H. spontaneum	L	MT334732	.	A	.	.	A	.	A	G	.	.	.
H. spontaneum	L	MT334733	.	A	.	.	A	.	A	G	.	.	.
H. spontaneum	L	MT334734	.	A	.	.	A	.	A	G	.	.	.
H. spontaneum	L	MT334735	.	A	.	.	A	.	A	G	.	.	.
H. spontaneum	L	MT334736	.	.	.	.	A	.	A	G	.	.	.

Germplasm	L189P	NCBI ref	A379T	L382Q	Q402H	K403M	I416V	V436I	N438S	T463A	R481M	Q482E	V490L
			G1136A	T1145A	G1206C	A1208T	A1248G	G1306A	A1404G	A1384G	A1439T	G1441C	G1465C
H. spontaneum	L	MT334737	.	.	.	.	A	.	A	G	.	.	.
H. spontaneum	L	MT334738	.	.	.	.	A	.	A	G	.	.	.
H. spontaneum	L	MT334739	.	.	.	.	A	.	.	G	.	.	.
H. spontaneum	L	MT334740	.	.	.	.	A	.	.	G	.	.	.
H. spontaneum	L	MT334741	.	.	.	.	A	.	.	G	.	.	.
H. spontaneum	L	MT334742	.	.	.	.	A	.	A	G	.	.	.
H. spontaneum	L	MT334743	.	.	.	.	A	.	A	G	.	.	.
H. spontaneum	L	MT334744	.	.	.	.	A	.	A	G	.	.	.
H. spontaneum	L	MT334745	.	.	.	.	A	.	A	G	.	.	.
H. spontaneum	L	MT334746	.	.	.	.	A	.	A	G	.	.	.
H. spontaneum	L	MT334747	.	.	.	.	A	.	A	G	.	.	.

H. spontaneum	L	MT334748	.	.	.	.	A	.	A	G	.	.	.
H. spontaneum	L	MT334749	.	.	.	.	A	.	A	G	.	.	.
H. spontaneum	L	MT334750	.	.	.	.	A	.	A	G	.	.	.
H. spontaneum	L	MT334751	.	.	.	.	A	.	A	G	.	.	.
H. spontaneum	L	MT334752	.	.	.	.	A	.	A	G	.	.	.
H. spontaneum	L	MT334753	.	.	.	.	A	.	A	G	.	.	.
H. spontaneum	L	MT334754	.	.	.	.	A	.	A	G	.	.	.
H. spontaneum	L	MT334755	.	.	.	.	A	.	A	G	.	.	.
H. spontaneum	L	MT334756	.	.	.	.	A	.	A	G	.	.	.
H. spontaneum	L	MT334757	.	.	.	.	A	.	A	G	.	.	.
H. spontaneum	L	MT334758	.	.	.	T	A	.	.	G	.	.	.
H. spontaneum	L	MT334759	.	.	.	T	A	.	.	G	.	.	.
H. spontaneum	L	MT334760	.	.	.	T	A	.	.	G	.	.	.
H. spontaneum	L	MT334761	.	.	.	T	A	.	.	G	.	.	.
H. spontaneum	L	MT334762	.	.	.	T	A	.	.	G	.	.	.
H. spontaneum	L	MT334763	.	.	.	T	A	.	.	G	.	.	.
H. spontaneum	L	MT334764	.	.	.	T	A	.	.	G	.	.	.
H. spontaneum	L	MT334765	.	.	.	T	A	.	.	G	.	.	.
H. spontaneum	L	MT334766	.	.	.	T	A	.	.	G	.	.	.
H. spontaneum	L	MT334767	.	.	.	T	A	.	.	G	.	.	.
H. spontaneum	L	MT334768	G	.	G	.	A	.	A	G	.	.	.
H. spontaneum	L	MT334769	G	.	G	.	A	.	A	G	.	.	.
H. spontaneum	L	MT334770	G	.	G	.	A	.	A	G	.	.	.
H. spontaneum	L	MT334771	G	.	G	.	A	.	A	G	.	.	.
H. spontaneum	L	MT334772	G	.	G	.	A	.	A	G	T	C	.
H. spontaneum	L	MT334773	.	A	.	.	A	.	A	G	.	.	.
H. spontaneum	L	MT334774	.	A	.	.	A	.	A	G	.	.	.
H. spontaneum	L	MT334775	G	.	G	.	A	.	A	G	.	.	.

Germplasm	L189P	NCBI ref	H502Y	R508T	V510L
			C1501T	C1520G	G1525C
H. spontaneum	P	MT334696	C	G	G
H. spontaneum	L	MT334697	.	.	.
H. spontaneum	L	MT334698	.	.	.
H. spontaneum	L	MT334699	.	.	C
H. spontaneum	L	MT334700	.	.	.
H. spontaneum	L	MT334701	.	.	.
H. spontaneum	L	MT334702	.	.	.
H. spontaneum	L	MT334703	.	.	.
H. spontaneum	L	MT334704	.	C	.
H. spontaneum	L	MT334705	.	C	.
H. spontaneum	L	MT334706	.	C	.
H. spontaneum	L	MT334707	.	C	.
H. spontaneum	L	MT334708	T	.	C
H. spontaneum	L	MT334709	.	C	.
H. spontaneum	L	MT334710	.	.	.
H. spontaneum	L	MT334711	.	.	.
H. spontaneum	L	MT334712	.	.	.
H. spontaneum	L	MT334713	.	.	.
H. spontaneum	L	MT334714	.	.	.
H. spontaneum	L	MT334715	.	.	.
H. spontaneum	L	MT334716	.	.	.
H. spontaneum	L	MT334717	.	.	.
H. spontaneum	L	MT334718	.	.	.
H. spontaneum	L	MT334719	.	.	.
H. spontaneum	L	MT334720	.	.	.
H. spontaneum	L	MT334721	.	.	.

H. spontaneum	L	MT334722	.	.	.
H. spontaneum	L	MT334723	.	.	.
H. spontaneum	L	MT334724	.	.	.
H. spontaneum	L	MT334725	.	.	.
H. spontaneum	L	MT334726	.	.	.
H. spontaneum	L	MT334727	T	.	C
H. spontaneum	L	MT334728	T	.	C
H. spontaneum	L	MT334729	T	.	C
H. spontaneum	L	MT334730	.	.	.
H. spontaneum	L	MT334731	.	.	.
H. spontaneum	L	MT334732	.	.	.
H. spontaneum	L	MT334733	.	.	.
H. spontaneum	L	MT334734	.	.	.
H. spontaneum	L	MT334735	.	.	.
H. spontaneum	L	MT334736	.	.	.

Germplasm	L189P	NCBI ref	H502Y	R508T	V510L
			C1501T	C1520G	G1525C
H. spontaneum	L	MT334737	.	.	.
H. spontaneum	L	MT334738	.	.	.
H. spontaneum	L	MT334739	T	.	C
H. spontaneum	L	MT334740	T	.	C
H. spontaneum	L	MT334741	T	.	C
H. spontaneum	L	MT334742	T	.	C
H. spontaneum	L	MT334743	.	.	.
H. spontaneum	L	MT334744	.	.	.
H. spontaneum	L	MT334745	.	.	.
H. spontaneum	L	MT334746	.	.	.
H. spontaneum	L	MT334747	.	.	.

H. spontaneum	L	MT334748	.	.	.
H. spontaneum	L	MT334749	.	.	.
H. spontaneum	L	MT334750	.	.	.
H. spontaneum	L	MT334751	.	.	.
H. spontaneum	L	MT334752	.	.	.
H. spontaneum	L	MT334753	.	.	.
H. spontaneum	L	MT334754	.	.	.
H. spontaneum	L	MT334755	.	.	.
H. spontaneum	L	MT334756	.	.	.
H. spontaneum	L	MT334757	.	.	.
H. spontaneum	L	MT334758	.	.	.
H. spontaneum	L	MT334759	.	C	.
H. spontaneum	L	MT334760	.	C	.
H. spontaneum	L	MT334761	.	C	.
H. spontaneum	L	MT334762	.	C	.
H. spontaneum	L	MT334763	.	C	.
H. spontaneum	L	MT334764	.	C	.
H. spontaneum	L	MT334765	.	C	.
H. spontaneum	L	MT334766	.	C	.
H. spontaneum	L	MT334767	.	.	.
H. spontaneum	L	MT334768	.	.	.
H. spontaneum	L	MT334769	.	.	.
H. spontaneum	L	MT334770	.	.	.
H. spontaneum	L	MT334771	.	.	.
H. spontaneum	L	MT334772	.	.	.
H. spontaneum	L	MT334773	.	.	.
H. spontaneum	L	MT334774	.	.	.
H. spontaneum	L	MT334775	.	C	.

Supplementary Table 4: Primers used for sanger sequencing, qPCR and In-situs

Application	Name	Sequence	Size	T_a (°C)
Sanger sequencin	HvHKT1;5_1F	AGCCACTTGTCAGTCGTAG	899	55
	HvHKT1;5_1R	CTGGTCCTTGAACCCTTGCT		
	HvHKT1;5_3F	CCTGATCTTCACGTCGGTGT	859	55
	HvHKT1;5_3R	CCTCGAGTTGACCGACATGA		
	HvHKT1;5_5F	CTCCTCGGAAACACGCTCT	869	55
	HvHKT1;5_5R	TTCCCTTCCCTGCTCCACTT		
	HvHKT1;5_7F	GCACAAGCTTCTCATGTCG	682	55
	HvHKT1;5_7R	GAGCCCGCTTACCTTCTCTC		
qPCR	HvHKT1;5_Q_2F	GCAGATCTCCGATGACCCA	175	65
	HvHKT1;5_Q_2R	TGAGCCTGCCGTAGAACATG		
In-situ	Insitu_HvHKT1;5_F	TGGTCATGATGTACCTACCA		
	Insitu_HvHKT1;5_R	GTACGCACTGATAACCTCGA		
	Insitu_Hv18s_F	ATGGCTCATTAAATCAGTTAT		
	Insitu_Hv18s_R	AATATACGCTATTGGAGCTGG		

Supplementary Table 5: Na+ contents of 5th leaf material from 0mM, 150mM and 250mM NaCl treated plants.

HvHKT1;5 haplotype	Cultivar Name	Treatment	Mean Na (mg/kg)
Na^+_{HAP1}	Golden Promise	0 mM NaCl	574.4
Na^+_{HAP2}	Viivi	0 mM NaCl	423.3
Na^+_{HAP3}	Morex	0 mM NaCl	470.3
Na^+_{HAP1}	Golden Promise	150 mM NaCl	3601.4
Na^+_{HAP2}	Viivi	150 mM NaCl	3201.9
Na^+_{HAP3}	Morex	150 mM NaCl	7677.0
Na^+_{HAP1}	Golden Promise	250 mM NaCl	6953.1
Na^+_{HAP2}	Viivi	250 mM NaCl	5555.8
Na^+_{HAP3}	Morex	250 mM NaCl	11054.3

Supplementary dataset: Influence of growth in 0mM, 150mM and 250mM on a range of phenotypic traits

Biomass and Grain Data set sample numbers

Treatment

Treatment	Biomass Data Freq	Grain Data Freq
Control	39	31
150 mM NaCl	40	28
250 mM NaCl	38	23

Allele, haplotype and line

Allele	Haplotype	Line	Freq Biomass Data	Freq Grain Data
L	Hap1	Aapo	15	11
L	Hap1	GP	15	12
L	Hap1	Rasa	15	8
L	Hap1	Tyne	15	9
L	Hap2	Viivi	15	10
P	Hap3	Ida	14	10
P	Hap3	Maris_Mink	15	13
P	Hap3	Ruja	13	9

Shoot data set sample numbers

Treatment

Treatment	Freq
Control	21
150 mM NaCl	20
250 mM NaCl	20

Line by Allele

Allele	Line	Freq
L	Golden_promise	20
L	Viivi	21
P	Morex	20

Analysis of phenotypic traits

There is confounding between Allele (2 levels), Haplotype (3 levels) and Line (8 levels) as one particular line can only have a single allele and haplotype-so essentially there are only 8 combinations of allele, haplotype and line. When fitting a model it is therefore not possible to fit a crossed interaction between these 3 factors as many of the combinations do not exist within the data set. The treatment structure for these 3 factors needs to be nested: Allele/Haplotypes/Lines. So lines are within haplotypes, which are within alleles.

The linear model approach presented aims to isolate whether the differences in trait are attributable to the alleles, haplotypes within alleles or lines within haplotypes within alleles or their interactions.

Salt treatment is also of interest, therefore a nested treatment structure between factors Allele, Haplotypes and Lines crossed with the salt Treatment can be used. The terms in the model are as follows:

Terms in the model

Term	Interpretation	df
(Intercept)	Overall mean	1
Allele	Allele	1
Treatment	Treatment	2
Allele:Haplotype	Haplotype	1
Allele:Treatment	Allele:Treatment	2
Allele:Haplotype:Line	Line	5
Allele:Haplotype:Treatment	Haplotype:Treatment	2
Allele:Haplotype:Line:Treatment	Line:Treatment	10

For the shoot Na, only three lines were included in the experiment, therefore Haplotype and Line are completely confounded and only one can be included in the model. The terms in the model are as follows:

Terms in the model

Term	Interpretation	df
(Intercept)	Overall Mean	1
Allele	Allele	1
Treatment	Treatment	2
Allele:Haplotype	Haplotype	1
Allele:Treatment	Allele:Treatment	2
Allele:Haplotype:Treatment	Haplotype:Treatment	2

Significance was tested at the 5% level. In general, terms in a model should be tested in a hierarchical manner, however, because of the confounding in this model there are exceptions to this. For example, the three-way interaction Allele:Haplotype:Treatment and the two way interaction Allele:Treatment can be examined simultaneously with the 4-way interaction. In addition, because of confounding there is an alternative interpretation of some terms. For example, the three-way interaction between Allele, Haplotype and Line can be interpreted as a line effect and the two way interaction between Allele and Haplotype corresponds to haplotype effects. Because of the difficult in interpretation of relevant significant terms, in the Anova tables below, terms highlighted are the appropriate significant terms for interpretation.

ASReml-R was which uses a REML approach was used for analysis because of the unbalanced nature of the data. Anova tables presented are from ASReml. For some lower order terms predicted values were not available from ASReml due to missing treatment combinations. For these terms predicted values were obtained using a linear regression model in Genstat. For consistency, if lower order terms were significant, all predicted values available are from a linear regression model conducted in Genstat. Differences between treatment levels was determined by Tukeys if the standard error of difference variance covariance matrix was available or by a Bonferonni corrected LSD (least significant difference) if just the average standard error of difference was available.

Biomass

There is a significant three-way interaction between Allele, Haplotype and Line and therefore the trait differs between lines. There is a significant interaction between Allele and Haplotype (borderline) and there is also a main effect of Treatment and of Allele. The treatment effect is independent of allele, haplotype and line. All predicted values are from Genstat regression.

Anova Table: Biomass

Term	Df	Sum of Sq	Wald statistic	Pr(Chisq)
Allele	1	17.3170719	10.3121835	0.0013215
Treatment	2	240.7291349	143.3523542	0.0000000
Allele:Haplotype	1	6.5975704	3.9288026	0.0474657
Allele:Treatment	2	3.3946952	2.0215150	0.3639432
Allele:Haplotype:Line	5	22.2350352	13.2407930	0.0212238
Allele:Haplotype:Treatment	2	0.2980298	0.1774745	0.9150860
Allele:Haplotype:Line:Treatment	10	17.4613580	10.3981048	0.4062873

Predicted Values (Allele by Haplotype by) Line: Biomass

Allele	Haplotype	Line	Predicted value	Std error	Group*
L	Hap1	Aapo	7.518	0.33467	abc
L	Hap1	GP	7.243	0.33467	ab
L	Hap1	Rasa	6.823	0.33467	a
L	Hap1	Tyne	6.688	0.33467	a
L	Hap2	Viivi	7.807	0.33467	abc
P	Hap3	Ida	7.125	0.34765	ab
P	Hap3	Maris_Mink	8.195	0.33467	bc
P	Hap3	Ruja	8.508	0.36082	b

**predicted mean values compared with bonferonni corrected LSD*

Predicted Values Treatment: Biomass

Treatment	Predicted value	Std error	Group*
Control	7.518	0.2297	a
150 mM NaCl	8.814	0.2290	b
250 mM NaCl	5.423	0.2306	c

**predicted mean values compared with bonferonni corrected LSD*

Predicted Values Haplotype: Biomass

Allele	Haplotype	Predicted value	Std error	Group*
L	Hap1	7.068	0.1673	a
L	Hap2	7.807	0.3347	b
P	Hap3	7.935	0.2005	b

**predicted mean values compared by bonferonni corrected LSD*

Predicted Values Allele: Biomass

Allele	Predicted value	Std error
L	7.111	0.2557
P	7.935	0.2557

Ear weight

There is a significant four-way interaction between Allele, Haplotype, Line and Treatment. Therefore the ear weight depends on the line and on the treatment.

Anova Table: Ear weight

Term	Df	Sum of Sq	Wald statistic	Pr(Chisq)
Allele	1	0.5417924	0.2075091	0.6487268
Treatment	2	95.5848289	36.6094558	0.0000000
Allele:Haplotype	1	9.0859323	3.4799564	0.0621164
Allele:Treatment	2	10.9303008	4.1863585	0.1232945
Allele:Haplotype:Line	5	44.8262946	17.1686895	0.0041907
Allele:Haplotype:Treatment	2	5.4976098	2.1056114	0.3489573
Allele:Haplotype:Line:Treatment	10	65.9813122	25.2711645	0.0048545

Predicted Values (Allele by Haplotype by) Line by Treatment: Ear weight

Allele	Haplotype	Line	Treatment	Predicted value	Std error	Group*
L	Hap1	Aapo	Control	4.03220	0.7226248	abcd
L	Hap1	Aapo	150 mM NaCl	3.95380	0.7226248	abcd
L	Hap1	Aapo	250 mM NaCl	2.16000	0.7226248	abc
L	Hap1	GP	Control	1.61380	0.7226248	ab
L	Hap1	GP	150 mM NaCl	5.43260	0.7226248	bcd
L	Hap1	GP	250 mM NaCl	2.52100	0.7226248	abc
L	Hap1	Rasa	Control	3.64140	0.7226248	abcd
L	Hap1	Rasa	150 mM NaCl	5.45900	0.7226248	cd
L	Hap1	Rasa	250 mM NaCl	3.47240	0.7226248	abcd
L	Hap1	Tyne	Control	6.37020	0.7226248	d
L	Hap1	Tyne	150 mM NaCl	6.42920	0.7226248	d

L	Hap1	Tyne	250 mM NaCl	2.75220	0.7226248	abcd
L	Hap2	Viivi	Control	3.99380	0.7226248	abcd
L	Hap2	Viivi	150 mM NaCl	3.86900	0.7226248	abcd
L	Hap2	Viivi	250 mM NaCl	1.48620	0.7226248	a
P	Hap3	Ida	Control	5.26840	0.7226248	abcd
P	Hap3	Ida	150 mM NaCl	3.04540	0.7226248	abcd
P	Hap3	Ida	250 mM NaCl	2.42975	0.8079191	abcd
P	Hap3	Maris_Mink	Control	4.39020	0.7226248	abcd
P	Hap3	Maris_Mink	150 mM NaCl	5.74300	0.7226248	cd
P	Hap3	Maris_Mink	250 mM NaCl	2.30740	0.7226248	abc
P	Hap3	Ruja	Control	3.22350	0.8079191	abcd
P	Hap3	Ruja	150 mM NaCl	3.17660	0.7226248	abcd
P	Hap3	Ruja	250 mM NaCl	2.97425	0.8079191	abcd

**predicted mean values compared with tukeys*

Biomass Combined with ear weight

There is a significant four-way interaction between Allele, Haplotype, Line and Treatment. Therefore the biomass combined with ear weight depends on both the line and the treatment with a non-additive interaction.

Anova Table: Biomass Combined with ear weight

Term	Df	Sum of Sq	Wald statistic	Pr(Chisq)
Allele	1	1.173277e+01	2.6159346	0.1057952
Treatment	2	6.374063e+02	142.1159035	0.0000000
Allele:Haplotype	1	1.986613e-01	0.0442935	0.8333086
Allele:Treatment	2	9.229523e+00	2.0578115	0.3573978
Allele:Haplotype:Line	5	3.632991e+01	8.1001046	0.1508043

Allele:Haplotype:Treatment	2	7.982469e+00	1.7797688	0.4107032
Allele:Haplotype:Line:Treatment	10	9.648986e+01	21.5133476	0.0177851

Predicted Values (Allele by Haplotype by) line by treatment: Biomass Combined with ear weight

Allele	Haplotype	Line	Treatment	Predicted value	Std error	Group*
L	Hap1	Aapo	Control	11.51860	0.9471131	abcdef
L	Hap1	Aapo	150 mM NaCl	12.97100	0.9471131	bcdef
L	Hap1	Aapo	250 mM NaCl	8.13340	0.9471131	ab
L	Hap1	GP	Control	8.80900	0.9471131	abcd
L	Hap1	GP	150 mM NaCl	14.23520	0.9471131	ef
L	Hap1	GP	250 mM NaCl	8.17160	0.9471131	ab
L	Hap1	Rasa	Control	10.81800	0.9471131	abcde
L	Hap1	Rasa	150 mM NaCl	13.76160	0.9471131	def
L	Hap1	Rasa	250 mM NaCl	8.37360	0.9471131	ab
L	Hap1	Tyne	Control	13.49560	0.9471131	cdef
L	Hap1	Tyne	150 mM NaCl	14.54280	0.9471131	ef
L	Hap1	Tyne	250 mM NaCl	7.49040	0.9471131	a
L	Hap2	Viivi	Control	12.15540	0.9471131	abcdef
L	Hap2	Viivi	150 mM NaCl	12.96200	0.9471131	bcdef
L	Hap2	Viivi	250 mM NaCl	7.57680	0.9471131	a
P	Hap3	Ida	Control	13.04420	0.9471131	bcdef
P	Hap3	Ida	150 mM NaCl	11.01880	0.9471131	abcde
P	Hap3	Ida	250 mM NaCl	7.99300	1.0589046	ab
P	Hap3	Maris_Mink	Control	12.35120	0.9471131	abcdef

P	Hap3	Maris_Mink	150 mM NaCl	16.14020	0.9471131	f
P	Hap3	Maris_Mink	250 mM NaCl	8.42520	0.9471131	ab
P	Hap3	Ruja	Control	12.69000	1.0589046	abcdef
P	Hap3	Ruja	150 mM NaCl	13.86180	0.9471131	ef
P	Hap3	Ruja	250 mM NaCl	8.20725	1.0589046	abc

**predicted mean values compared with tukeys*

TGW

There is a significant three-way interaction between Allele, Haplotype and Line and between Allele and Treatment.

Anova Table: TGW

Term	Df	Sum of Sq	Wald statistic	Pr(Chisq)
Allele	1	10.34065	0.1274180	0.7211243
Treatment	2	558.85998	6.8863022	0.0319638
Allele:Haplotype	1	126.85402	1.5631019	0.2112116
Allele:Treatment	2	986.99781	12.1618390	0.0022861
Allele:Haplotype:Line	5	1190.09690	14.6644368	0.0118966
Allele:Haplotype:Treatment	2	21.45482	0.2643674	0.8761800
Allele:Haplotype:Line:Treatment	10	1202.71240	14.8198857	0.1387678

Predicted Values (Allele by Haplotype by) Line: TGW

Allele	Haplotype	Line	Predicted value	Std error	Group*
L	Hap1	Aapo	40.433	2.3265	b
L	Hap1	GP	30.658	2.3265	a
L	Hap1	Rasa	38.521	2.3265	b
L	Hap1	Tyne	34.419	2.3265	ab
L	Hap2	Viivi	39.285	2.3265	b
P	Hap3	Ida	36.752	2.4168	ab
P	Hap3	Maris_Mink	32.606	2.3265	ab

P	Hap3	Ruja	39.577	2.5083	b

**predicted mean values compared with corrected bonferonni LSD*

Predicted Values Allele by Treatment: TGW

Allele	Treatment	Predicted value	Std error	Group*
L	Control	33.13	1.911	a
L	150 mM NaCl	42.02	1.911	a
L	250 mM NaCl	33.23	1.911	a
P	Control	35.69	2.412	a
P	150 mM NaCl	33.96	2.330	a
P	250 mM NaCl	38.91	2.504	a

**predicted mean values compared with bonferonni corrected LSD*

Area

There is a significant three-way interaction between Allele, Haplotype and Line and the two-way interaction between Allele and Treatment effect and between Allele and Haplotype.

Anova Table: Area Anova Table: Area

Term	Df	Sum of Sq	Wald statistic	Pr(Chisq)
Allele	1	3.895407e-01	8.517500e-02	0.7704031
Treatment	2	9.862775e+00	2.156545e+00	0.3401826
Allele:Haplotype	1	1.276616e+02	2.791386e+01	0.0000001
Allele:Treatment	2	3.302763e+01	7.221657e+00	0.0270294
Allele:Haplotype:Line	5	9.551324e+01	2.088445e+01	0.0008518
Allele:Haplotype:Treatment	2	2.041867e+00	4.464644e-01	0.7999291
Allele:Haplotype:Line:Treatment	10	5.998659e+01	1.311637e+01	0.2172380

Predicted Values (Allele by Haplotype by) Line: Area

Allele	Haplotype	Line	Predicted value	Std error	Group*
L	Hap1	Aapo	22.604	0.55229	cd
L	Hap1	GP	20.159	0.55229	a
L	Hap1	Rasa	21.296	0.55229	abc
L	Hap1	Tyne	20.097	0.55229	a

L	Hap2	Viivi	24.292	0.55229	d
P	Hap3	Ida	22.180	0.57372	bc
P	Hap3	Maris_Mink	20.680	0.55229	ab
P	Hap3	Ruja	22.797	0.59545	cd

**predicted mean values compared with bonferonni corrected LSD*

Predicted Values Allele by Treatment: Area

Allele	Treatment	Predicted value	Std error	Group*
L	Control	20.84	0.4536	a
L	150 mM NaCl	22.20	0.4536	a
L	250 mM NaCl	20.61	0.4536	a
P	Control	21.67	0.5726	a
P	150 mM NaCl	21.31	0.5531	a
P	250 mM NaCl	22.56	0.5943	a

**predicted mean values compared with bonferonni corrected LSD*

Predicted Values (Allele by) Haplotype: Area

Allele	Haplotype	Predicted value	Std error	Group*
L	Hap1	21.04	0.2761	a
L	Hap2	24.29	0.5523	b
P	Hap3	21.84	0.3308	a

**predicted mean values compared with bonferonni corrected LSD*

Width

There is a significant interaction between allele and treatment.

Anova Table: Width

Term	Df	Sum of Sq	Wald statistic	Pr(Chisq)
Allele	1	0.0265934	3.090325e-01	0.5782745
Treatment	2	0.1189233	1.381965e+00	0.5010836
Allele:Haplotype	1	0.0833333	9.683869e-01	0.3250828
Allele:Treatment	2	0.5880284	6.833268e+00	0.0328227

Allele:Haplotype:Line	5	0.8446874	9.815810e+00	0.0806256
Allele:Haplotype:Treatment	2	0.0330667	3.842559e-01	0.8252013
Allele:Haplotype:Line:Treatment	10	1.2746752	1.481255e+01	0.1390471

Predicted Values Allele by Treatment: Width

Allele	Treatment	Predicted value	Std error	Group*
L	Control	3.136	0.06222	a
L	150 mM NaCl	3.311	0.06222	a
L	250 mM NaCl	3.098	0.06222	a
P	Control	3.194	0.07854	a
P	150 mM NaCl	3.083	0.07587	a
P	250 mM NaCl	3.230	0.08153	a

**predicted mean values compared with bonferonni corrected LSD*

Length

There is significant three-way interactions between Allele, Haplotype and Treatment and between Allele, Haplotype and Line.

Anova Table: Length

Term	Df	Sum of Sq	Wald statistic	Pr(Chisq)
Allele	1	0.1047680	6.900687e-01	0.4061410
Treatment	2	0.0501037	3.300145e-01	0.8478876
Allele:Haplotype	1	13.0208333	8.576348e+01	0.0000000
Allele:Treatment	2	0.1207161	7.951131e-01	0.6719599
Allele:Haplotype:Line	5	3.1422507	2.069686e+01	0.0009241
Allele:Haplotype:Treatment	2	0.9760667	6.428995e+00	0.0401755
Allele:Haplotype:Line:Treatment	10	0.8202914	5.402960e+00	0.8626875

Predicted Values (Allele by Haplotype by) Line: Length

Allele	Haplotype	Line	Predicted value	Std error	Group*
L	Hap1	Aapo	8.768	0.10063	c

L	Hap1	GP	8.339	0.10063	a
L	Hap1	Rasa	8.389	0.10063	ab
L	Hap1	Tyne	8.236	0.10063	a
L	Hap2	Viivi	9.470	0.10063	d
P	Hap3	Ida	8.864	0.10453	c
P	Hap3	Maris_Mink	8.565	0.10063	abc
P	Hap3	Ruja	8.683	0.10849	abc

**predicted mean values compared with bonferonni corrected LSD*

Predicted Values (Allele by) Haplotype by Treatment: Length

Allele	Haplotype	Treatment	Predicted value	Std error	Group
L	Hap1	Control	8.460	0.08713	a
L	Hap1	150 mM NaCl	8.500	0.08713	a
L	Hap1	250 mM NaCl	8.335	0.08713	a
L	Hap2	Control	9.300	0.17425	b
L	Hap2	150 mM NaCl	9.340	0.17425	b
L	Hap2	250 mM NaCl	9.780	0.17425	b
P	Hap3	Control	8.634	0.10432	a
P	Hap3	150 mM NaCl	8.687	0.10078	a
P	Hap3	250 mM NaCl	8.786	0.10829	a

**predicted mean values compared with bonferonni corrected LSD*

Grain Na

A square root transformations for Grain Na was necessary in order to meet model assumptions. There is a significant four way interaction between Allele, Haplotype, Line and treatment. Therefore the Grain Na changes depending on the line and treatment. There is also a significant two-way interaction between Allele and Treatment and between Allele and Haplotype

Anova Table: Grain Na

Term	Df	Sum of Sq	Wald statistic	Pr(Chisq)
Allele	1	5801.32847	134.599678	0.0000000
Treatment	2	1293.23865	30.005111	0.0000003
Allele:Haplotype	1	213.27734	4.948360	0.0261154
Allele:Treatment	2	1034.18945	23.994774	0.0000062
Allele:Haplotype:Line	5	2587.21671	60.027378	0.0000000
Allele:Haplotype:Treatment	2	50.30663	1.167191	0.5578890
Allele:Haplotype:Line:Treatment	10	1549.98297	35.961971	0.0000855

Back Transformed Predicted Values for (Allele by Haplotype by) Line by treatment: Grain Na

Allele	Haplotype	Line	Treatment	Predicted value	Std error	groups	Back transformed predicted value	Back transformed Std error
L	Hap1	Aapo	Control	14.48421	2.936005	ab	209.7922	85.05139
L	Hap1	Aapo	150 mM NaCl	18.68596	3.790366	ab	349.1649	141.65320
L	Hap1	Aapo	250 mM NaCl	17.69716	3.790366	ab	313.1894	134.15740
L	Hap1	GP	Control	17.31248	2.936005	ab	299.7220	101.65905
L	Hap1	GP	150 mM NaCl	20.73275	3.282553	ab	429.8469	136.11270
L	Hap1	GP	250 mM NaCl	23.69988	3.790366	ab	561.6842	179.66240
L	Hap1	Rasa	Control	15.40053	3.790366	ab	237.1763	116.74727
L	Hap1	Rasa	150 mM NaCl	15.34165	3.790366	ab	235.3663	116.30093
L	Hap1	Rasa	250 mM NaCl	13.27139	4.642231	ab	176.1297	123.21767
L	Hap1	Tyne	Control	15.97358	3.282553	ab	255.1552	104.86823

L	Hap1	Tyne	150 mM NaCl	18.89766	3.790366	ab	357.1216	143.25809
L	Hap1	Tyne	250 mM NaCl	24.83871	4.642231	ab	616.9616	230.61405
L	Hap2	Viivi	Control	13.93784	3.790366	ab	194.2633	105.65900
L	Hap2	Viivi	150 mM NaCl	10.49707	3.790366	a	110.1884	79.57545
L	Hap2	Viivi	250 mM NaCl	16.06363	3.282553	ab	258.0403	105.45944
P	Hap3	Ida	Control	22.64528	3.790366	ab	512.8089	171.66780
P	Hap3	Ida	150 mM NaCl	31.14192	3.282553	b	969.8190	204.44997
P	Hap3	Ida	250 mM NaCl	27.02554	3.790366	ab	730.3798	204.87335
P	Hap3	Marris Mink	Control	22.82041	2.936005	ab	520.7712	134.00166
P	Hap3	Marris Mink	150 mM NaCl	53.42992	2.936005	c	2854.7563	313.74097
P	Hap3	Marris Mink	250 mM NaCl	63.33185	3.790366	c	4010.9238	480.10175
P	Hap3	Ruja	Control	21.99550	3.790366	ab	483.8020	166.74196
P	Hap3	Ruja	150 mM NaCl	30.50674	3.790366	ab	930.6611	231.26338
P	Hap3	Ruja	250 mM NaCl	30.88116	3.790366	ab	953.6458	234.10174

**predicted mean values on transformed scale compared with tukeys*

Back Transformed Predicted Values for (Allele by) Treatment: Grain Na

Allele	Treatment	Predicted value	Std error	Group*	Back transformed predicted value	Back transformed Std error
L	Control	15.74	1.519	a	247.75	47.82

L	150 mM NaCl	18.20	1.739	a	331.24	63.30
L	250 mM NaCl	19.98	1.969	a	399.20	78.68
P	Control	22.53	1.991	a	507.60	89.71
P	150 mM NaCl	40.02	1.900	b	1601.60	152.08
P	250 mM NaCl	42.86	2.216	b	1836.98	189.96

** predicted mean values on transformed scale compared with bonferonni corrected LSD*

Back Transformed Predicted Values for (Allele by) Haplotype: Grain Na

Allele	Haplotype	Predicted value	Std error	Group*	Back transformed predicted value	Back transformed Std error
L	Hap1	18.04	1.0472	a	325.44	37.78
L	Hap2	13.36	2.1392	b	178.49	57.16
P	Hap3	34.20	1.1720	c	1169.64	80.16

**predicted mean values on transformed scale compared with bonferonni corrected LSD*

Grain K

There is a significant treatment effect. Allele, Haplotype and Line are not significant.

Anova Table: Grain K

Term	Df	Sum of Sq	Wald statistic	Pr(Chisq)
Allele	1	2.639048e+06	2.4878311	0.1147297
Treatment	2	8.649643e+06	8.1540218	0.0169581
Allele:Haplotype	1	5.751935e+05	0.5422351	0.4615079
Allele:Treatment	2	5.369355e+03	0.0050617	0.9974724
Allele:Haplotype:Line	5	7.656622e+06	7.2179002	0.2049326
Allele:Haplotype:Treatment	2	1.365310e+06	1.2870778	0.5254297
Allele:Haplotype:Line:Treatment	10	7.635811e+06	7.1982817	0.7066027

Predicted Values treatment: Grain K

Treatment	Predicted value	Std error	Group*
Control	7751	192.7	a
150 mM NaCl	7329	213.7	ab
250 mM NaCl	7077	243.0	b

** predicted mean values of salt treatment compared to control with LSD.*

Shoot Na

A square root transformations for Grain Na was necessary in order to meet model assumptions. There is a significant two-way interaction between Allele and Treatment. Therefore, the response differs depending on both the allele and treatment.

Anova Table: Shoot Na

Term	Df	Sum of Sq	Wald statistic	Pr(Chisq)
Allele	1	7056.94885	63.6587791	0.0000000
Treatment	2	44749.94004	403.6768024	0.0000000
Allele:Haplotype	1	280.59661	2.5311842	0.1116164
Allele:Treatment	2	2462.70965	22.2154209	0.0000150
Allele:Haplotype:Treatment	2	65.64216	0.5921397	0.7437355

Back Transformed Predicted Values for Allele by Treatment: Shoot Na

Allele	Treatment	Predicted value	Std error	Group*	Back transformed predicted value	Back transformed Std error
L	Control	21.77	2.721	a	473.93	118.47
L	150 mM NaCl	57.01	2.924	b	3250.14	333.39
L	250 mM NaCl	77.64	2.935	c	6027.97	455.75
P	Control	21.51	4.298	a	462.68	184.90
P	150 mM NaCl	87.44	3.980	c	7645.75	696.02

P	250 mM NaCl	104.56	3.980	d	10932.79	832.30

**predicted mean values on transformed scale compared with bonferonni corrected LSD*
