# Supplementary material for: Barley sodium content is regulated by natural variants of the Na+ transporter HvHKT1;5
Source: Commun Biol. 2020 May 22;3:258. doi: 10.1038/s42003-020-0990-5 (PMC7244711; doi:10.1038/s42003-020-0990-5)
Supplement: Supplementary file 2 — Description of Additional Supplementary Items [file 42003_2020_990_MOESM2_ESM.pdf]

Description of additional supplementary items

All 5 supplementary data files are included in the Excel file.

**Supplementary Data 1:**	Elite 2-row spring cultivars included in GWAS and sequenced for *HvHKT1*;5
**Supplementary Data 2:**	Gene models in region identified on 4H as being significantly associated with grain Na^+ content
**Supplementary Data 3:**	*H. spontaneum* and *H. vulgare* landrace *HvHKT1;5* genotypic data
**Supplementary Data 4:**	Primers used for Sanger sequencing, qPCR and In-situs.
**Supplementary Data 5:**	Na^+ contents of 5th leaf material from 0mM, 150mM and 250mM NaCl treated plants.
