# Supplementary material for: Barley sodium content is regulated by natural variants of the Na+ transporter HvHKT1;5
Source: Commun Biol. 2020 May 22;3:258. doi: 10.1038/s42003-020-0990-5 (PMC7244711; doi:10.1038/s42003-020-0990-5)
Supplement: Supplementary file 4 — Reporting Summary [file 42003_2020_990_MOESM4_ESM.pdf]

nature research

Corresponding author(s): Robbie Waugh

Last updated by author(s): 4/28/2020

Reporting Summary

Nature Research wishes to improve the reproducibility of the work that we publish. This form provides structure for consistency and transparency in reporting. For further information on Nature Research policies, seeAuthors & Referees Editorial Policy Checklist

Statistics

For all statistical analyses, confirm that the following items are present in the figure legend, table legend, main text, or Methods section.

n/a	Confirmed	
☐	☒	The exact sample size (n
☐	☒	A statement on whether measurements were taken from distinct samples or whether the same sample was measured repeatedly
☐	☒	The statistical test(s) used AND whether they are one- or two-sided<br>*Only common tests should be described solely by name; describe more complex techniques in the Methods section.*
☐	☒	A description of all covariates tested
☐	☒	A description of any assumptions or corrections, such as tests of normality and adjustment for multiple comparisons
☐	☒	A full description of the statistical parameters including central tendency (e.g. means) or other basic estimates (e.g. regression coefficient) AND variation (e.g. standard deviation) or associated estimates of uncertainty (e.g. confidence intervals)
☐	☒	For null hypothesis testing, the test statistic (e.g. F t r P<br>*Give P values as exact values whenever suitable.*
☒	☐	For Bayesian analysis, information on the choice of priors and Markov chain Monte Carlo settings
☐	☒	For hierarchical and complex designs, identification of the appropriate level for tests and full reporting of outcomes
☒	☐	Estimates of effect sizes (e.g. Cohen's d r

Our web collection on statistics for biologists

Software and code

Policy information about availability of computer code

Data collection	This does not apply to the current study
Data analysis	This does not apply to the current study

For manuscripts utilizing custom algorithms or software that are central to the research but not yet described in published literature, software must be made available to editors/reviewers. We strongly encourage code deposition in a community repository (e.g. GitHub). See the Nature Research guidelines for submitting code & software

Data

Policy information about availability of data

All manuscripts must include a data availability statement

- Accession codes, unique identifiers, or web links for publicly available datasets
- A list of figures that have associated raw data
- A description of any restrictions on data availability

NCBI accession numbers for Sanger sequence data corresponding to HvHKT1;5 are available in Supplementary Tables 1 and 3. Genotyping data used for GWAS and all other relevant data not provided in this manuscript already are available from the corresponding authors upon request.

Field-specific reporting

Please select the one below that is the best fit for your research. If you are not sure, read the appropriate sections before making your selection.

☒ Life sciences ☐ Behavioural & social sciences ☐ Ecological, evolutionary & environmental sciences

Life sciences study design

All studies must disclose on these points even when the disclosure is negative.

Sample size	Information provided in Methods. We used 131 contemporary European 2-rowed spring barley genotypes for the GWAS panel and resequencing. The L189P SNP was genotyped in 184 georeferenced genotypes.<br><br>For screening leaf and grain Na+ accumulation under salt stress conditions, we used 8 barley genotypes and 3 germinated seeds per genotype per pot (8 replicate pots). Seedlings thinned to 1 after emergence of 2nd leaf.
Data exclusions	Information provided in Methods. For the phylogenetic analysis, sites containing gaps in more than 5% of the data were removed from the analysis.
Replication	Information provided in Methods. See Sample size section above.
Randomization	Did not use
Blinding	Did not use

Reporting for specific materials, systems and methods

We require information from authors about some types of materials, experimental systems and methods used in many studies. Here, indicate whether each material, system or method listed is relevant to your study. If you are not sure if a list item applies to your research, read the appropriate section before selecting a response.

Materials & experimental systems

n/a	Involved in the study
☒	☐ Antibodies
☒	☐ Eukaryotic cell lines
☒	☐ Palaeontology
☒	☐ Animals and other organisms
☒	☐ Human research participants
☒	☐ Clinical data

Methods

n/a	Involved in the study
☒	☐ ChIP-seq
☒	☐ Flow cytometry
☒	☐ MRI-based neuroimaging
